# An immunometabolic patch facilitates mesenchymal stromal/stem cell therapy for myocardial infarction through a macrophage‐dependent mechanism

**DOI:** 10.1002/btm2.10471

**Published:** 2022-12-13

**Authors:** Weizhang Xiao, Ming Chen, Wenjing Zhou, Liang Ding, Ziying Yang, Lianbo Shao, Jingjing Li, Weiqian Chen, Zhenya Shen

**Affiliations:** ^1^ Department of Cardiovascular Surgery of the First Affiliated Hospital & Institute for Cardiovascular Science Suzhou Medical College of Soochow University Suzhou China; ^2^ Department of Cardiothoracic Surgery Affiliated Hospital and Medical School of Nantong University Nantong China

**Keywords:** glycolysis, immunometabolism, macrophage, mesenchymal stromal/stem cells, myocardial infarction, patch

## Abstract

Mesenchymal stromal/stem cells (MSCs) have emerged as a promising approach against myocardial infarction. Due to hostile hyperinflammation, however, poor retention of transplanted cells seriously impedes their clinical applications. Proinflammatory M1 macrophages, which rely on glycolysis as their main energy source, aggravate hyperinflammatory response and cardiac injury in ischemic region. Here, we showed that the administration of an inhibitor of glycolysis, 2‐deoxy‐d‐glucose (2‐DG), blocked the hyperinflammatory response within the ischemic myocardium and subsequently extended effective retention of transplanted MSCs. Mechanistically, 2‐DG blocked the proinflammatory polarization of macrophages and suppressed the production of inflammatory cytokines. Selective macrophage depletion abrogated this curative effect. Finally, to avoid potential organ toxicity caused by systemic inhibition of glycolysis, we developed a novel chitosan/gelatin‐based 2‐DG patch that directly adhered to the infarcted region and facilitated MSC‐mediated cardiac healing with undetectable side effects. This study pioneered the application of an immunometabolic patch in MSC‐based therapy and provided insights into the therapeutic mechanism and advantages of this innovative biomaterial.

## 1 INTRODUCTION

Myocardial infarction (MI), which is principally caused by the stenosis and occlusion of coronary arteries, remains the leading cause of most acute cardiac events and deaths worldwide.^1^ Many studies and clinical trials have supported the efficacy of mesenchymal stromal/stem cells (MSCs) in patients with MI.^2^, ^3^ Our previous studies have explored whether MSC therapy reduces scarring, minimizes perfusion defects, and ameliorates cardiac dysfunction in animal MI models.^4^, ^5^, ^6^ Nonetheless, MSC application faces serious obstacles in terms of the low retention and survival of implanted cells, which are attributed to hostile hyperinflammation in the ischemic region. After MI attack, glycolysis occurs in the infarct area due to hypoxia and exhausted nutrients, accompanied by the accumulation of acid metabolites, free radicals, necrotic cells, and degraded extracellular matrix, which provoke sterile inflammation and the inflammatory activation of immune cells.^7^, ^8^ Previous studies have determined the crucial role of macrophages in cardiac injury and established the heterogeneity of macrophages during the process of cardiac injury and repair.^9^ Several hours after MI, monocytes are recruited to the infarct zone and differentiate into proinflammatory M1 macrophages. These macrophage subpopulations initially predominate and culminate on Day 3, which generates a cascade of cytokines to trigger intense inflammatory responses and injury.^10^ Then, the number of M1 macrophages decreases and these cells are replaced by reparative M2 macrophages, which represent the predominant subset after 5 days.^11^ Although M1 macrophages function positively to clear cellular debris, their excessive activities inevitably extend the duration of inflammation and undermine cardiac recovery.^12^ To date, treatments targeting M1 macrophages or promoting the M1/M2 switch have been considered novel strategies for MI.^13^, ^14^ However, few studies have scrutinized the immunomodulatory effect of metabolism on macrophages.

The main energy source of M1 macrophages is glycolysis, whereas M2 macrophages rely largely on oxidative phosphorylation.^15^ Reprogrammed immunometabolism in macrophages contributes to the modulation of the inflammatory response.^16^ As shown in our previous report, curbing the aspartate‐arginosuccinate shunt, which compensates for the fragmented tricarboxylic acid cycle during glycolysis, ameliorates cardiac function in a murine MI model through immunometabolic reprogramming of macrophages.^17^ As a classical glycolytic inhibitor, 2‐deoxy‐D‐glucose (2‐DG) has long been proved to effectively suppress the growth of tumor cells, the energy production of which depends mainly on aerobic glycolysis.^18^, ^19^ However, the steeply increased glycolysis after MI occurs in a confined ischemic area, and researchers have not determined whether 2‐DG exerts an immunometabolic modulation on the local hyperinflammation and contributes to cardiac protection against MI.

In this study, we proposed an immunomodulatory effect of 2‐DG on macrophages through the repression of glycolysis. By detecting the functional switch of macrophages and the generation of proinflammatory factors, we explored the alteration of the inflammatory response in vivo after 2‐DG supplementation. Considering the pitfalls of systemic 2‐DG administration, including a limited half‐life and potential organ toxicity,^20^ local application of 2‐DG through a cardiac patch has entered our view. Chitosan and gelatin are two valuable biomaterials with their characteristics of biocompatibility, biodegradation, nontoxicity, and plasticity.^21^ Under acidic conditions, cation‐rich chitosan reacts with gelatin that contains anions to form polyelectrolyte complexes, which have been widely used in biomedical and tissue engineering applications including wound healing,^22^ drug delivery,^23^ and cardiac repair.^24^ Therefore, we developed an innovative chitosan/gelatin‐based 2‐DG patch (2‐DGp_at_) and investigated its synergistic effect on MSC‐based therapy by assessing the retention of implanted MSCs and subsequent cardiac recovery.

## 2 RESULTS

### 2.1 Effect of glycolytic inhibition on immunometabolism of macrophages

**FIGURE 1:**
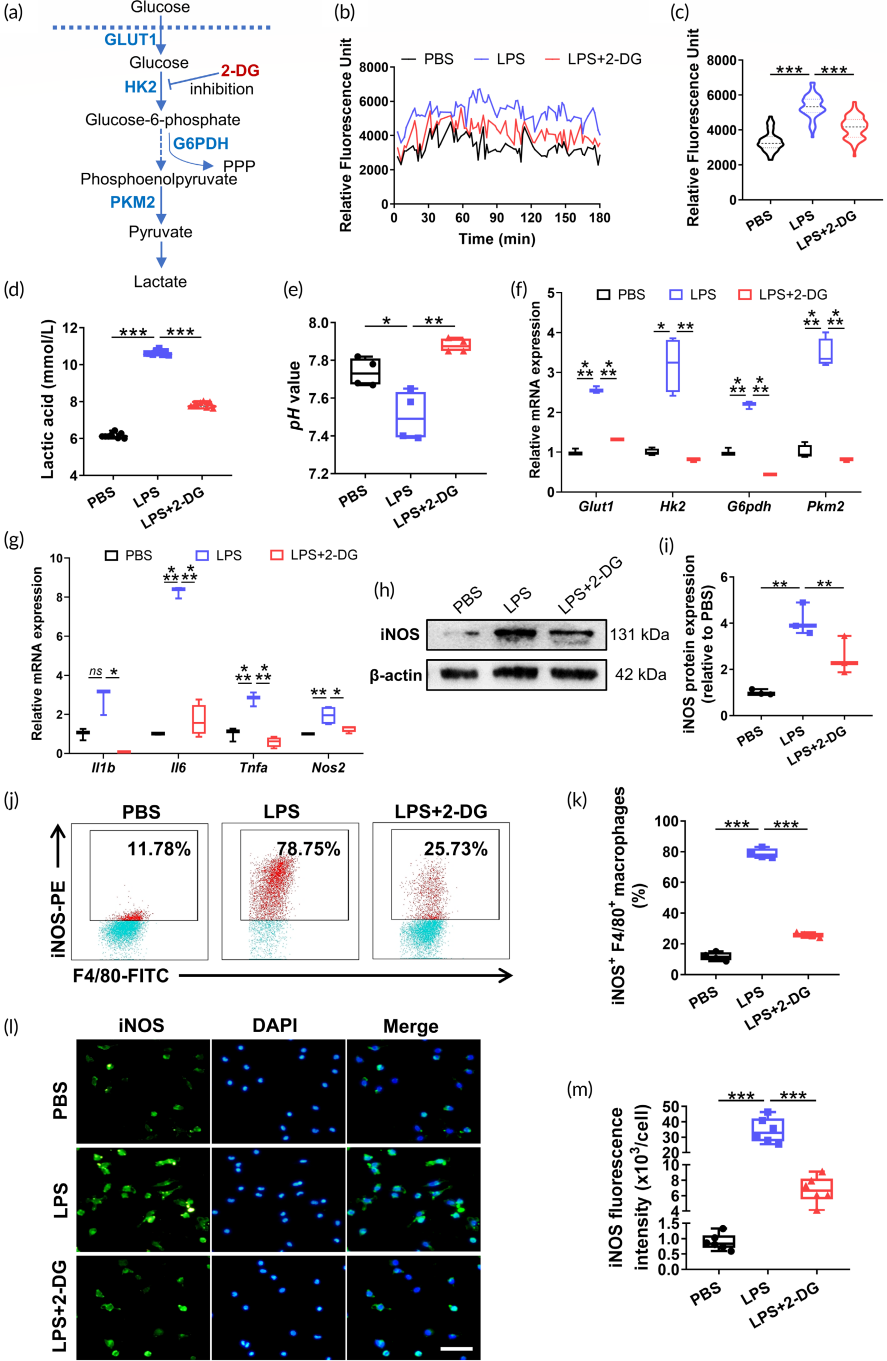
Glycolytic inhibition attenuates the proinflammatory polarization of macrophages. (a) Schematic representation of the glycolytic pathway. (b, c) Representative graph (b) and quantitative analysis (c) of the extracellular acidification rate (ECAR) assay (*n* = 3). (d) Lactate production by macrophages (*n* = 8). (e) pH values of macrophage supernatants (*n* = 4). (f) Relative expression of glycolytic rate‐limiting enzymes detected using real‐time PCR (*n* = 3–4). (g) Gene expression of proinflammatory factors related to M1 polarization (*n* = 3–4). (h, i) The protein levels of iNOS detected using western blotting (h) and the quantification of immunoblotting (i) (*n* = 3). (j, k) Representative flow cytometry plots (j) and quantification (k) of the iNOS^+^ macrophage population (*n* = 4). (l, m) Representative fluorescence images (l) and quantification of the iNOS (green) fluorescence intensity (m) (*n* = 6). Scale bar, 50 μm. Error bars represent the SD, and significance was determined using one‐way ANOVA followed by Tukey's test (*p < 0.05, **p < 0.01, and ***p < 0.001).

2‐DG is considered to efficiently blunt glycolysis by competitively restraining hexokinase 2 (HK2), which catalyzes the initial step of glucose metabolism (Figure 1a).^20^ Here, lipopolysaccharide (LPS)‐stimulated macrophages were pretreated with or without 2‐DG to elucidate the effect of glycolytic inhibition on immunophenotypic switch of macrophages. We initially analyzed the extracellular acidification rate (ECAR) in macrophages and detected an expectedly notable reversion of the LPS‐mediated increase in glycolytic flux after pretreatment with 2‐DG (Figure 1b,c). Meanwhile, 2‐DG supplementation decreased the lactate content in culture medium and subsequently increased the pH value (Figure 1d,e), confirming a suppressive effect of 2‐DG on glycolytic metabolism. Subsequently, we discovered that the LPS‐induced upregulation of several glycolytic rate‐limiting enzymes (Figure 1a), including glucose transporter 1 (Glut1), hexokinase 2 (Hk2), glucose 6‐phosphate dehydrogenase (G6pdh), and pyruvate kinase isoform M2 (Pkm2), was dramatically reversed by 2‐DG supplementation (Figure 1f).

We subsequently determined whether glucose metabolic reprogramming modulated the immunophenotypic switch of macrophages. As expected, when pretreated with 2‐DG, the production of inflammatory mediators was strikingly reduced at both the transcript (Figure 1g) and protein levels (Figure 1h,i). Moreover, as illustrated by flow cytometry and immunofluorescence staining, the 2‐DG pretreatment substantially reduced the iNOS^+^F4/80^+^ cell proportion from 78.75% ± 3.20% to 25.73% ± 1.49% (Figure 1j,k) and the inducible nitric oxide synthase (iNOS) fluorescence intensity (Figure 1l,m), reflecting the mitigated proinflammatory polarization of macrophages. Intriguingly, 2‐DG failed to modulate the CD206^+^ F4/80^+^ macrophages (*p* = 0.812) (Figure S1A,B), which was consistent with a previous study exploring the M1–M2 repolarization of inflammatory macrophages.^25^ Taken together, these results provide important insights into the role of 2‐DG in the immunomodulation of macrophages by inactivating glycolytic metabolism.

### 2.2 Therapeutic effects of systemic 2‐DG against hyperinflammation in ischemic myocardium

**FIGURE 2:**
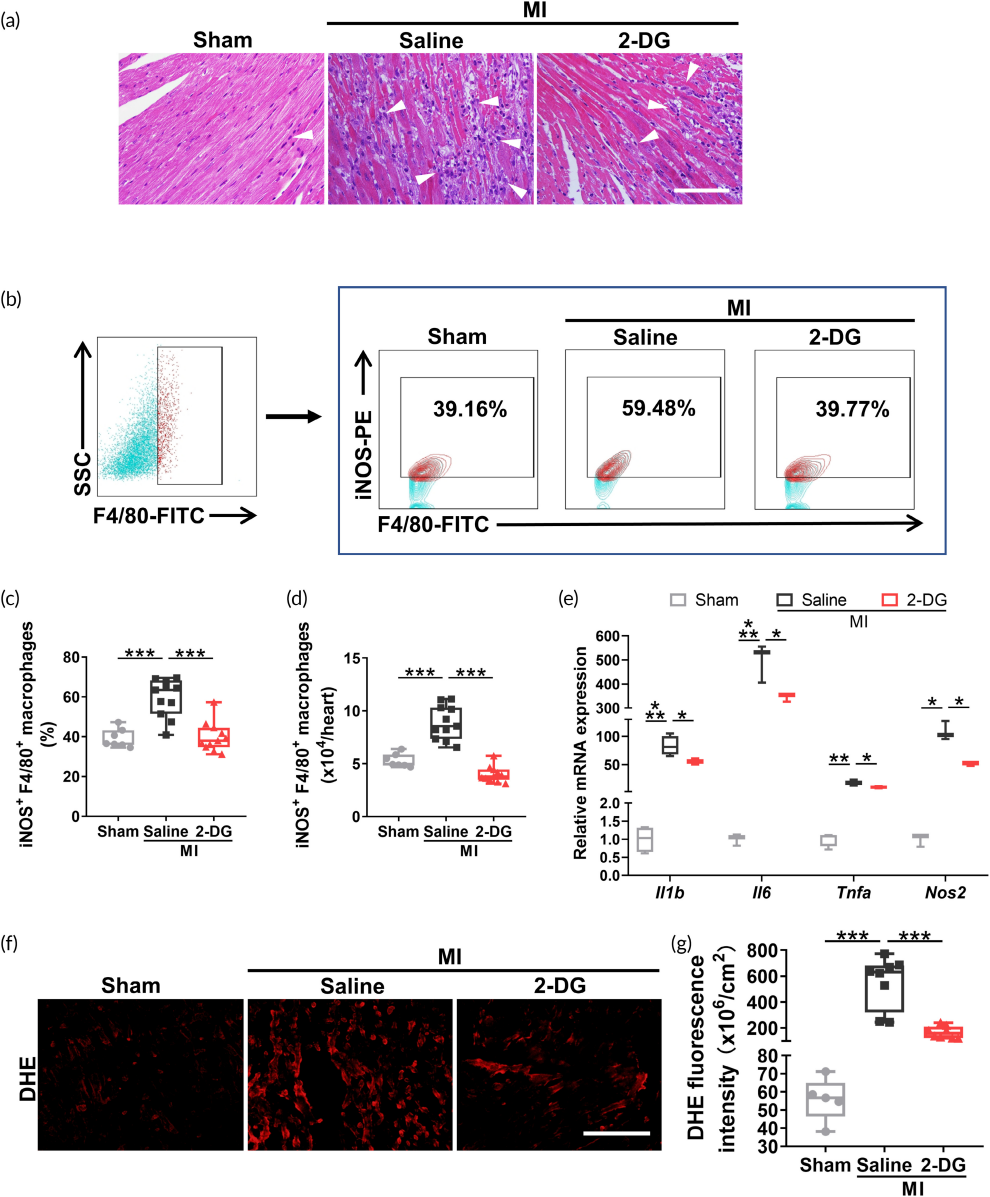
Decreased glycolysis attenuates the local hyperinflammatory response in ischemic hearts. (a) Representative image of hematoxylin and eosin (H&E) staining indicating inflammatory cells (white arrow) in heart sections collected 3 days after myocardial infarction (MI). Scale bar, 100 μm. (b) Representative flow cytometry plots of iNOS^+^F4/80^+^ macrophages in the infarcted area 3 days after MI. (c, d) Quantification of the percentage (c) and number (d) of iNOS^+^F4/80^+^ macrophages in (b) (*n* = 7–11). (e) Expression of the Il1β, Il6, Tnfα, and Nos2 mRNAs in the ischemic region detected using real‐time PCR analysis 3 days post‐MI (*n* = 3–4). (f) Representative graph of dihydroethidium (DHE) staining (red) indicating the superoxide levels in the infarct zone at 3 days after MI. Scale bar, 200 μm. (g) Quantification of the DHE fluorescence intensity (*n* = 5–8). Error bars represent the SD, and significance was determined using one‐way ANOVA followed by Tukey's test (*p < 0.05, **p < 0.01, and ***p < 0.001).

In order to determine whether 2‐DG treatment tempers detrimental inflammation in vivo, we intraperitoneally injected 2‐DG into MI mice and analyzed the inflammatory response within the infarcted area at 3 days after MI when the number of proinflammatory macrophages peaked. As illustrated by hematoxylin and eosin (H&E) staining, the accumulation of immune cells in the infarct zone was dramatically reduced by 2‐DG administration (Figure 2a). In particular, both proportion (39.77% ± 7.46% vs. 59.48% ± 9.56%, *p* < 0.001) and number of proinflammatory iNOS M1 macrophages were decreased in 2‐DG‐treated infarcted hearts (Figure 2b–d), consistent with the immune reprogramming of macrophages in vitro. However, 2‐DG treatment failed to increase the anti‐inflammatory CD206^+^ F4/80^+^ macrophages in ischemic area (*p* = 0.119) (Figure S2A,B). Due to the large decrease in M1 macrophages, 2‐DG altered the balance of M1/M2 macrophage phenotypes in vivo (Figure S2C).

Infarcted hearts were harvested to determine whether 2‐DG treatment modulates the inflammatory cytokine storm in the ischemic area. First, the mRNA levels of inflammatory factors were downregulated after the 2‐DG injection (Figure 2e). Meanwhile, the expression of rate‐limiting enzymes in the infarcted myocardium was evidently decreased in the 2‐DG group compared with the saline controls (Figure S3), indicating abrogated glycolysis. Moreover, excessive ROS accumulation occurs in the myocardium after MI and contributes to a hostile microenvironment, jeopardizing cell therapy. We therefore performed dihydroethidium (DHE) staining and revealed that 2‐DG supplementation inactivated MI‐induced superoxide generation (Figure 2f,g). Collectively, 2‐DG attenuates hyperinflammatory injury in the ischemic region.

### 2.3 2‐DG benefits cell retention and cardioprotective role of MSCs therapy

**FIGURE 3:**
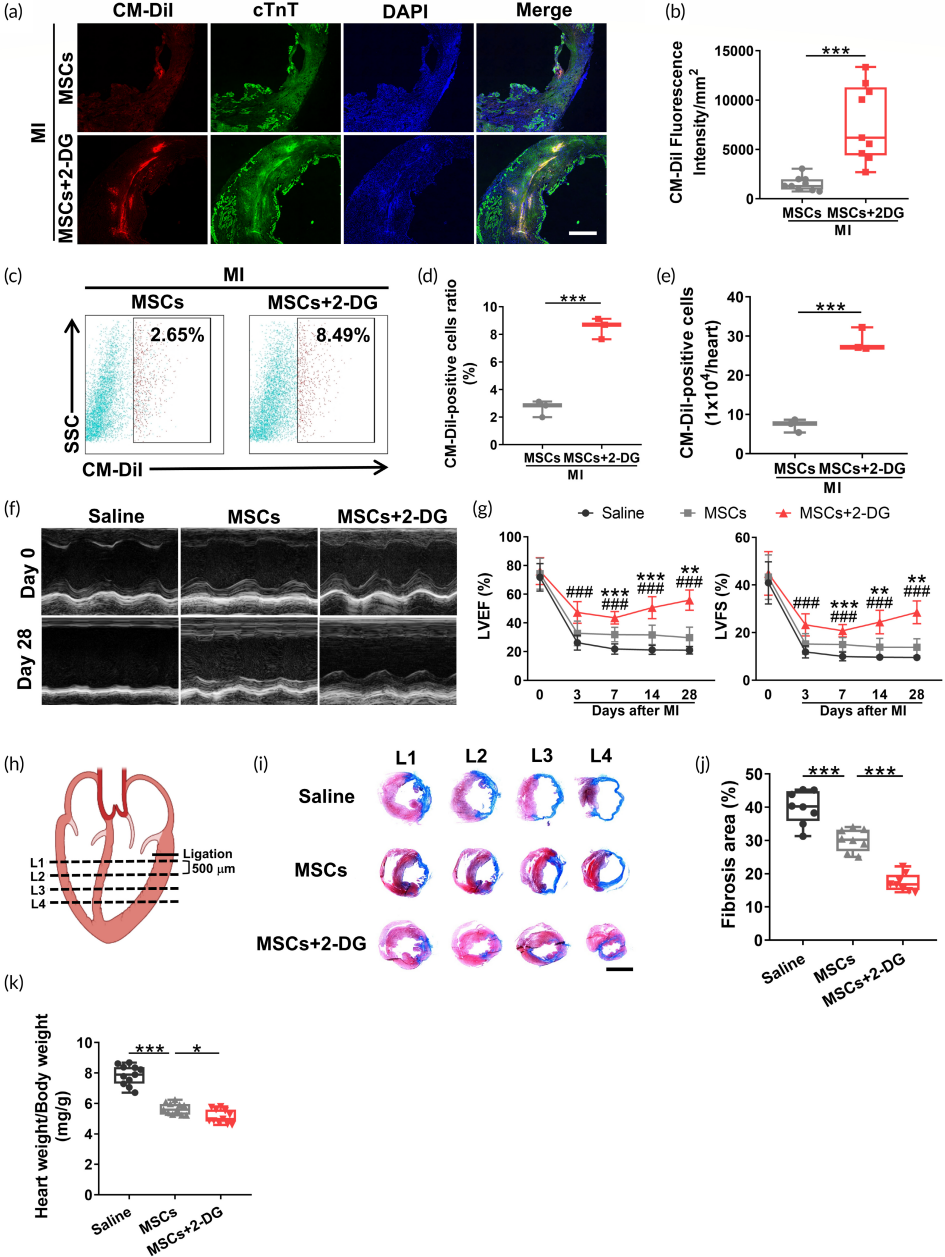
Limited glycolysis increases the efficacy of mesenchymal stromal/stem cells (MSC) therapy in myocardial infarction (MI)‐challenged mice. (a) Representative chloromethylbenzamido (CM‐DiI) (red) and cTnT (green) fluorescence images of infarcted hearts 3 days after cell transplantation. Scale bar, 500 μm. (b) Quantitative analysis of CM‐DiI fluorescence in the infarct zone (*n* = 8). (c) Representative flow cytometry plots of CM‐DiI‐positive cells on Day 3 post‐MI. (d, e) Quantification of the percentage (d) and number (e) of CM‐DiI‐positive cells in hearts (*n* = 3). (f) Representative short‐axis M‐mode echocardiography recordings of infarcted hearts on Days 0 and 28 post‐MI. (g) Quantitative analysis of left ventricular ejection fraction (LVEF) and left ventricular fractional shortening (LVFS) on Days 0, 3, 7, 14, and 28 after MI (*n* = 11–12). * for the comparison of the MSCs group with the saline group; # for the comparison of the MSCs + 2‐DG group with the MSC group. (h) Schematic diagram of the slices collected at 500 μm intervals from the ligation plane to the apex of the heart. (i) Representative images of Masson's trichrome‐stained heart sections 28 days after surgery. Scale bar, 4 mm. (j) Quantification of the fibrotic area (*n* = 8). (k) Quantification of the heart weight/body weight ratio (*n* = 11). Data are presented as the means ± SD and were analyzed using one‐way ANOVA followed by Tukey's test. LVEF and LVFS were analyzed using two‐way ANOVA followed by Tukey's test (*p < 0.05, **p < 0.01, ***p < 0.001, #*p* < 0.05, and ##*p* < 0.01).

We next intended to verify whether 2‐DG may promote superior retention of MSCs in the ischemic myocardium. Murine bone marrow MSCs were first validated by their surface marker expression (Figure S4A) as well as adipogenic (Figure S4B), osteogenic (Figure S4C), and chondrogenic (Figure S4D) differentiation potential. Then, MSCs were prelabeled with 2 μg/ml chloromethylbenzamido (CM‐DiI), which allowed us to track the live cells in hearts after MI. On Day 3 after MI, hearts were harvested for ex vivo analysis. As illustrated in Figure 3a, clusters of CM‐DiI signals were detected in the infarcted area after MSCs implantation. More importantly, MSCs + 2‐DG group possesses more CM‐DiI signal in comparison with MSCs alone, demonstrating a promoted MSCs retention (*p* = 0.035) (Figure 3b). A subsequent flow cytometry analysis also revealed a pronounced increase in both the ratio (2.65% ± 0.59% vs. 8.49% ± 0.76%, *p* < 0.001) (Figure 3c,d) and number (Figure 3e) of CM‐DiI‐positive cells after 2‐DG supplementation, indicating enhanced retention of implanted MSCs.

Transthoracic echocardiography was conducted at consecutive time points after MI to evaluate whether 2‐DG amplifies MSC‐mediated cardiac repair. As illustrated in Figure 3f,g, the MI‐induced reduction of left ventricular ejection fraction (LVEF) and fractional shortening (LVFS) was partially reversed by MSCs implantation and was further recovered with greater significance by the addition of 2‐DG (LVEF: 55.88% ± 7.12% vs. 29.68% ± 7.37%, *p* < 0.001; LVFS: 28.51% ± 4.80% vs. 13.80% ± 3.62%, *p* < 0.001 on Day 28 post‐MI). Consistent with these findings, a decreased fibrotic area was observed in mice treated with the combination of MSCs + 2‐DG on Day 28 after surgery (Figure 3h–j). Furthermore, the morphological analysis revealed a diminished relative heart weight in MI‐challenged mice treated with the combination of MSCs + 2‐DG, representing tempered ventricular hypertrophy (Figure 3k).

Collectively, 2‐DG exerts a cardioprotective effect on myocardial injury by enhancing the retention of MSCs, which resist MI‐induced cardiac dysfunction.

### 2.4 2‐DG facilitates MSCs therapy in a macrophage‐dependent manner

**FIGURE 4:**
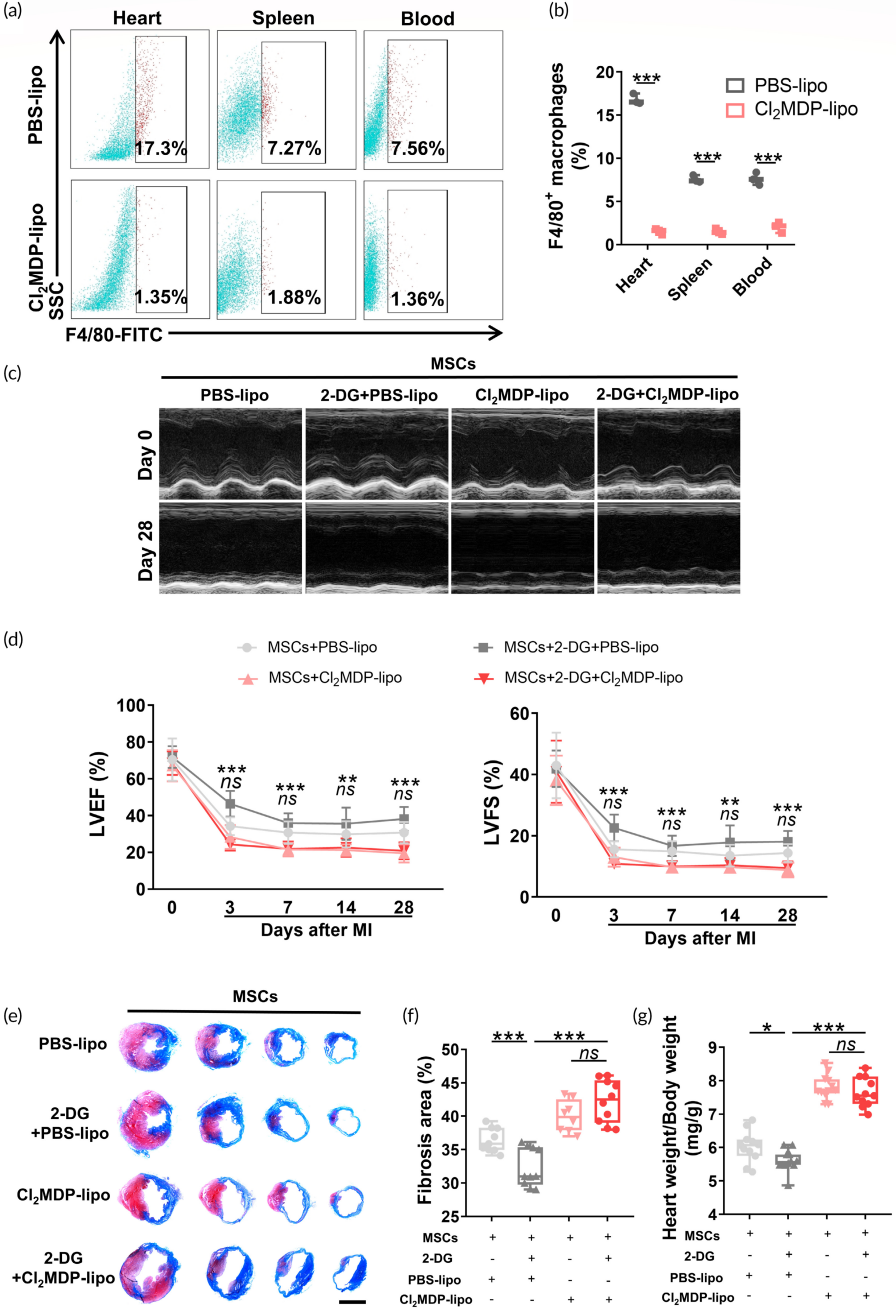
Systemic macrophage depletion obliterates the therapeutic effect of 2‐deoxy‐d‐glucose (2‐DG). (a, b) Representative flow cytometry plots (a) and pooled data (b) from F4/80^+^ macrophages in the hearts, spleens, and blood of PBS‐lipo‐ or Cl_2_MDP‐lipo‐treated mice 3 days after myocardial infarction (MI) (*n* = 3). (c) Representative short‐axis M‐mode echocardiography images on Days 0 and 28 post‐MI. (d) Quantitative analysis of the left ventricular ejection fraction (LVEF) and left ventricular fractional shortening (LVFS) (*n* = 11–13). * for the comparison of the MSCs + 2‐DG + Cl_2_MDP‐lipo group with the MSCs + 2‐DG + PBS‐lipo group; *ns*, not significant for the comparison of the MSCs + 2‐DG + Cl_2_MDP‐lipo group with the MSCs + Cl_2_MDP‐lipo group. (e) Representative graphs of Masson's trichrome staining in infarcted hearts 28 days after MI. Scale bar, 4 mm. (f) Quantification of the fibrotic area in (e) (*n* = 8–10). (g) Quantification of the heart weight/body weight ratio (*n* = 11–12). All data are presented as the means ± SD. Statistically significant differences were determined using one‐way ANOVA followed by Tukey's test. LVEF and LVFS were analyzed using two‐way ANOVA followed by Tukey's test (*p < 0.05, **p < 0.01, ***p < 0.001, and *ns* for not significant).

We selectively depleted macrophages by administering Cl_2_MDP to exclude the possibility that 2‐DG exerts its cardioprotective effect by modulating the immunometabolism of cells other than macrophages. Consistent with a previous report,^26^ Cl_2_MDP successfully removed macrophages from the heart, spleen, and blood (Figure 4a,b). Interestingly, 2‐DG delivery did not enable MSCs to resist MI‐induced cardiac dysfunction in macrophage‐depleted mice (LVEF: 20.82% ± 4.65% vs. 19.45% ± 5.02%, *p* = 0.926; LVFS: 9.47% ± 2.15% vs. 8.83% ± 2.42%, *p* = 0.927 on Day 28 post‐MI) (Figure 4c,d). Consistently, macrophage deficiency also abrogated the effect of 2‐DG on reducing the scar formation (Figure 4e,f) and ventricular remodeling (Figure 4g), suggesting that macrophages were required for the 2‐DG‐mediated cardioprotective benefits. Taken together, these results provide critical insights into the effect of selective obliteration of macrophages on compromising the benefits of 2‐DG treatment, insinuating that macrophages are indispensable for 2‐DG to facilitate MSC therapy.

### 2.5 2‐DG_pat_ exerts modulation of in vitro macrophage polarization

**FIGURE 5:**
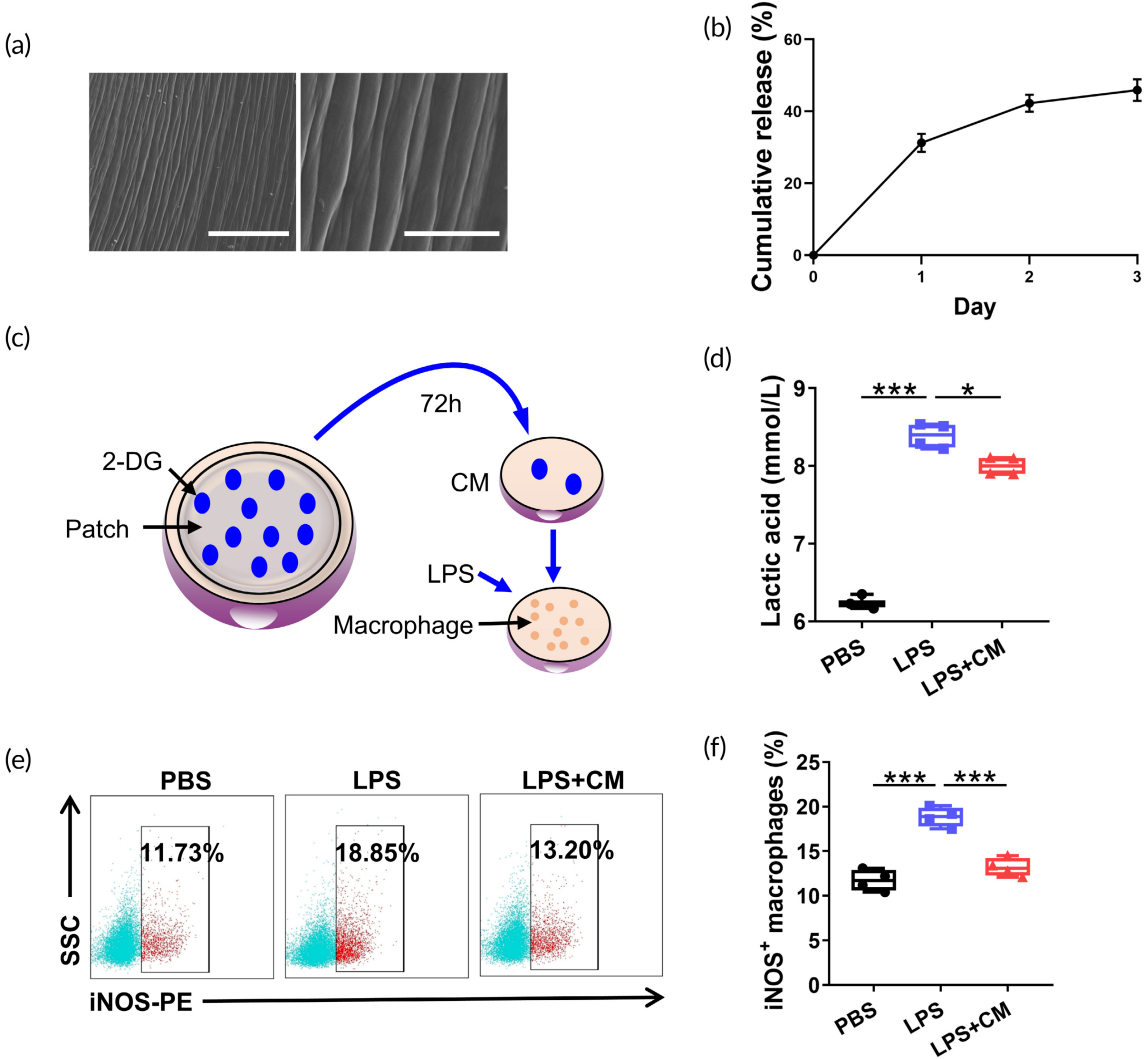
2‐Deoxy‐d‐glucose (2‐DG_pat_) regulates macrophage immunometabolism in vitro. (a) Visualization of 2‐DG_pat_ using scanning electron microscopy. Scale bar, left, 100 μm; right, 30 μm. (b) The cumulative release curve of 2‐DG_pat_. (c) Schematic representation of the method used to collect conditioned medium (CM) from 2‐DG_pat_ for further analysis. (d) Lactic acid production by macrophages incubated with CM with or without lipopolysaccharide (LPS) stimulation (*n* = 3–4). (e) Representative flow cytometry plots of the iNOS^+^ macrophage subpopulation. (f) Quantitative analysis of iNOS^+^ macrophages in (d) (*n* = 4). Error bars represent SD, and significance was determined using one‐way ANOVA followed by Tukey's test (*p < 0.05, **p < 0.01, ***p < 0.001, and *ns* for not significant).

Previous studies have highlighted several side effects related to systemic 2‐DG administration, such as fatigue, dizziness, nausea,^27^ body weight loss,^28^, ^29^ and even increased mortality^30^; therefore, we employed a chitosan/gelatin‐based 2‐DG patch to minimize potential side effects. As illustrated in Figure 5a, scanning electron microscopy revealed the 2‐DG_pat_ with a ravined and nonporous surface. To demonstrate the sustained release of 2‐DG from the patch, we detected the 2‐DG concentration in the supernatant of 2‐DG_pat_. As shown in Figure 5b, 2‐DG_pat_ continuously and slowly released 2‐DG into the surrounding supernatant, and the release rate was highest on the first day (>30%). After that, the release rate gradually decreased, and there was still a continuous release of 2‐DG on the third day. We subsequently investigated its biological properties in vitro to ensure that 2‐DG_pat_ elicits a similar therapeutic response in the ischemic myocardium. The medium containing 10% fetal bovine serum (FBS) was added to 2‐DG_pat_ and the conditioned medium (CM) was collected 72 h after initial soaking to treat macrophages (Figure 5c). The LPS‐mediated lactate production was partially reversed by the incubation of CM, unambiguously suggesting the restriction of myeloid glycolysis (Figure 5d). Not surprisingly, the presence of 2‐DG in CM significantly diminished the iNOS^+^ macrophage ratio (Figure 5e,f) compared to the LPS control, indicating the modulation of M1 polarization. Collectively, these results suggested that 2‐DG released from patches remained bioactive for at least 3 days.

### 2.6 2‐DG_pat_ extends in vivo retention of transplanted MSCs

**FIGURE 6:**
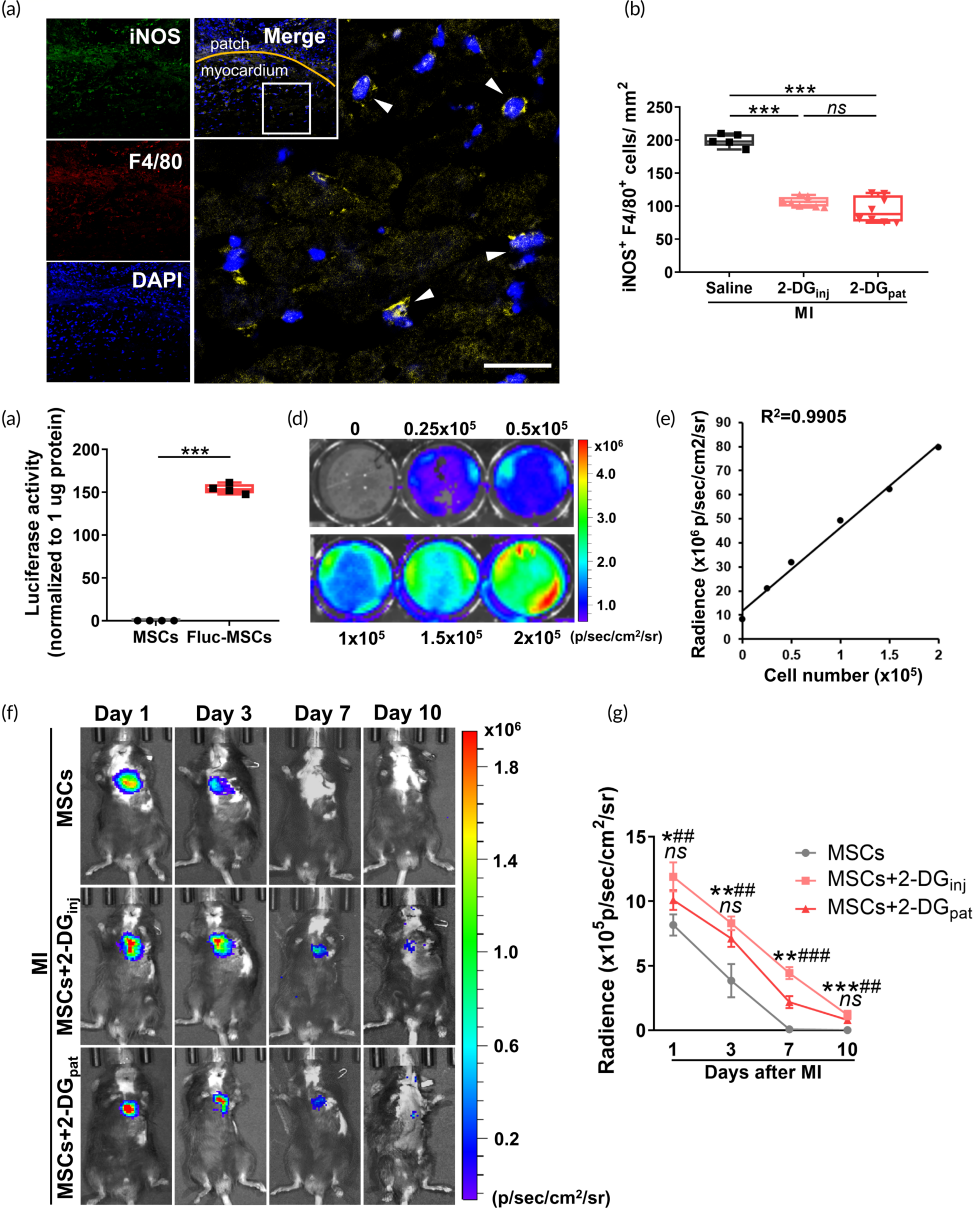
Immunometabolic patches promote the survival of transplanted mesenchymal stromal/stem cells (MSCs) by alleviating the local hyperinflammatory response after myocardial infarction (MI). (a) Representative confocal fluorescence imaging of iNOS (green)‐ and F4/80 (red)‐positive cells (white arrow) within the ischemic region of mice treated with 2‐DG_pat_ on Day 3 after MI. Scale bar, 50 μm. (b) Quantification of iNOS^+^ F4/80^+^ cells in the 2‐DG_pat_ group compared to the saline and 2‐DG_inj_ groups (*n* = 5–8). (c) Luciferase activity measured in MSCs and Fluc‐MSCs (*n* = 4). (d) Luciferase activity of Fluc‐MSCs with different cell numbers captured using the IVIS system. (e) Significant linear correlation between the cell number and bioluminescence imaging (BLI) signals (*R* ^2^ = 0.9905). (f) Representative graphs of in vivo BLI at 1, 3, 7, and 10 days after Fluc‐MSC transplantation. (g) Quantitative analysis of BLI signals in (f) (*n* = 5). * for the comparison of the MSCs + 2‐DG_pat_ group with the MSCs group; # for the comparison of the MSCs + 2‐DG_inj_ group with the MSCs group; *ns* equals to no significant difference between the MSCs + 2‐DG_pat_ group and MSCs + 2‐DG_inj_ group. All data are presented as the means ± SD, and significant differences were determined using ANOVA followed by Tukey's test (*p < 0.05, **p < 0.01, ***p < 0.001, #*p* < 0.05, and *ns* for not significant).

Based on these aforementioned promising in vitro observations, we next questioned whether local 2‐DG_pat_ generated a similar anti‐inflammatory effect as a systemic injection. A rounded 2‐DG_pat_ measuring 3.5 × 3.5 mm was attached onto the infarct area immediately after left anterior descending artery (LAD) ligation. Expectedly, fewer iNOS^+^F4/80^+^ cells in 2‐DG_pat_‐treated hearts 3 days after surgery were observed than in saline controls (Figure 6a,b). No discernable variation was observed between mice treated with 2‐DG_pat_ and those undergoing 2‐DG injection (*p* = 0.339) (Figure 6b).

We next assessed whether 2‐DG_pat_ prolonged retention of MSCs in the infarcted myocardium. A lentivirus containing firefly luciferase was transduced into MSCs (Fluc‐MSCs) for in vivo cell tracking. As illustrated in Figure 6c, Fluc‐MSCs exhibited more than 1000‐fold higher luciferase activity than MSCs. Meanwhile, in vitro bioluminescence imaging (BLI) revealed a positive linear correlation between the firefly luciferase activity and cell number (Figure 6d,e). Then, Fluc‐MSCs were injected into infarcted myocardium and tracked consecutively after MI. As the experiment proceeded, the myocardial BLI signal in all mice decreased substantially and almost completely disappeared 7 days post‐MI, indicating a substantial reduction in the number of implanted MSCs (Figure 6f). In contrast, both systemic and topical applications of 2‐DG effectively prolonged the retention of MSCs (Figure 6g). More importantly, no difference in BLI signal was observed between the MSCs + 2‐DG_pat_ and MSCs + 2‐DG_inj_ groups (*p* = 0.051 for Day 1, *p* = 0.130 for Day 3, and *p* = 0.142 for Day 10), suggesting comparable MSC retention (Figure 6g). In conclusion, consistent with the results obtained from the systemic injection, 2‐DG_pat_ also possesses the capability to promote the retention of implanted MSCs by suppressing hyperinflammation in infarcted hearts.

### 2.7 2‐DG_pat_ benefits cardiac recovery efficiency by MSCs therapy

**FIGURE 7:**
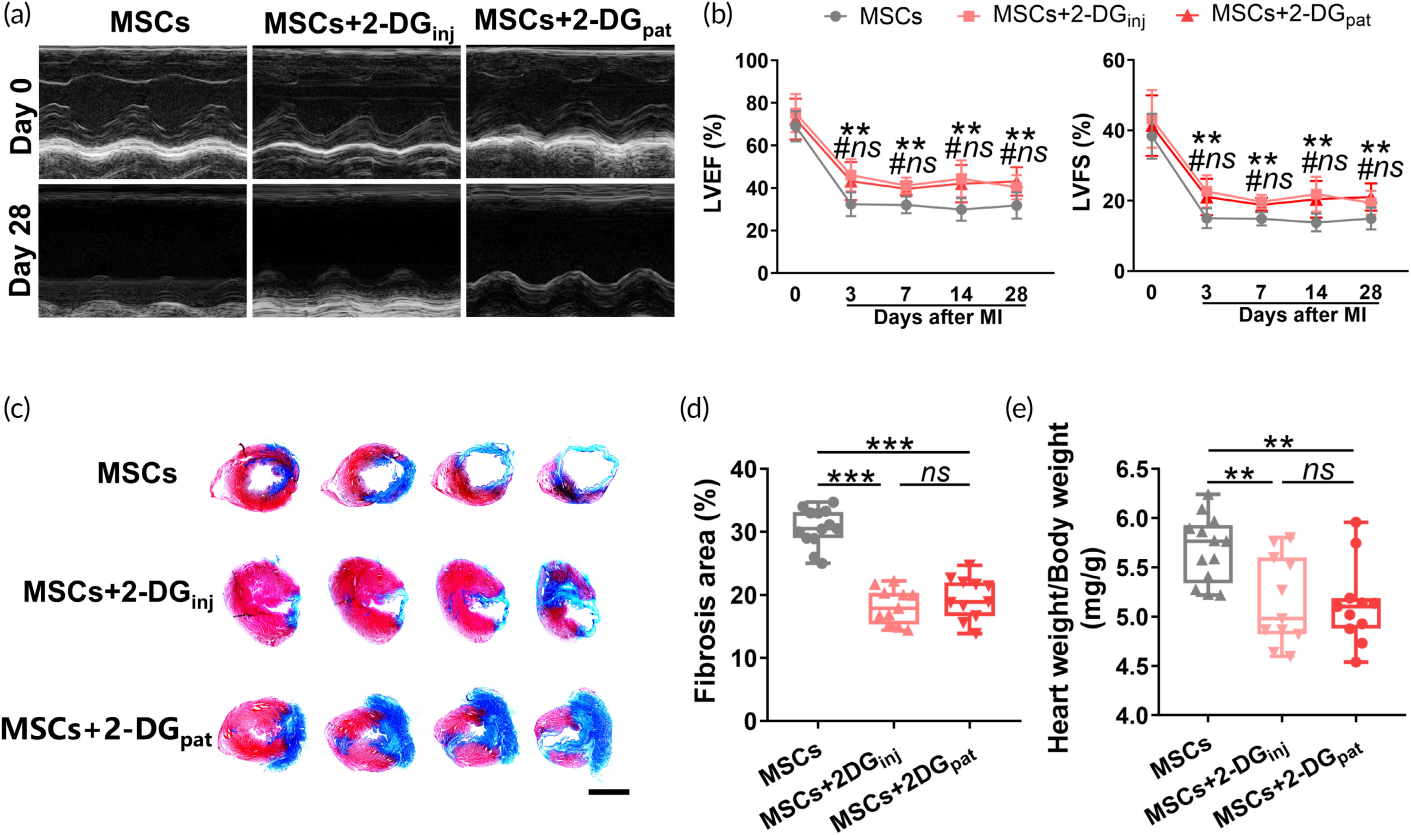
Immunometabolic patches exert similar cardioprotective effects on mice that experienced myocardial infarction (MI) as 2‐DG injections. (a) Representative short‐axis M‐mode echocardiography of hearts on Days 0 and 28 post‐MI. (b) Quantitative analysis of the left ventricular ejection fraction (LVEF) and left ventricular fractional shortening (LVFS) (*n* = 11–13). * for the comparison of the MSCs + 2‐DG_pat_ group with the MSC group; # for the comparison of the MSCs + 2‐DG_inj_ group with the MSC group; *ns* indicates no significant difference between the MSCs + 2‐DG_pat_ group and MSCs + 2‐DG_inj_ group. (c) Representative images of Masson's trichrome staining in infarcted hearts 28 days after MI. Scale bar, 4 mm. (d) Quantification of the fibrotic area in (c) (*n* = 11–13). (e) Quantification of the heart weight/body weight ratio (*n* = 11–13). All data are presented as the means ± SD. Statistically significant differences were determined using one‐way ANOVA followed by Tukey's test. Cardiac function was analyzed using two‐way ANOVA followed by Tukey's test (*p < 0.05, **p < 0.01, #*p* < 0.05, and *ns* for not significant).

We next evaluated the effect of 2‐DG_pat_ on post‐MI cardiac function using echocardiography. As illustrated in Figure 7a,b, compared with MSCs alone, mice in the MSCs + 2‐DG_pat_ group exhibited improved myocardial function even on Day 28 post‐MI (EF: 31.82% ± 6.16% vs. 43.08 %± 6.73%, *p* = 0.001; FS: 14.93% ± 3.13% vs. 21.06% ± 3.90%, *p* = 0.001). Moreover, no detectable difference was observed between the MSCs + 2‐DG_pat_ and MSCs + 2‐DG_inj_ groups (LVEF: 43.08% ± 6.73% vs. 40.36% ± 5.66%, *p* = 0.569; LVFS: 21.06% ± 3.90% vs. 19.44% ± 3.35%, *p* = 0.559), indicating comparable beneficial effects of 2‐DG_pat_ and 2‐DG_inj_ on MSC‐mediated cardiac recovery. The infarct size was subsequently calculated by performing Masson's trichrome staining on Day 28 post‐MI. Compared with MSC therapy alone, MSC + 2‐DG_pat_ therapy produced a smaller fibrotic area (Figure 7c,d) and relative heart weight (Figure 7e). Overall, these data support the hypothesis that 2‐DG_pat_ exerts a synergistic effect with MSC therapy on preventing adverse cardiac remodeling and preserving cardiac function, similar to 2‐DG_inj_.

### 2.8 2‐DG_pat_ facilitates MSCs‐mediated cardiac healing with limited side effects

**FIGURE 8:**
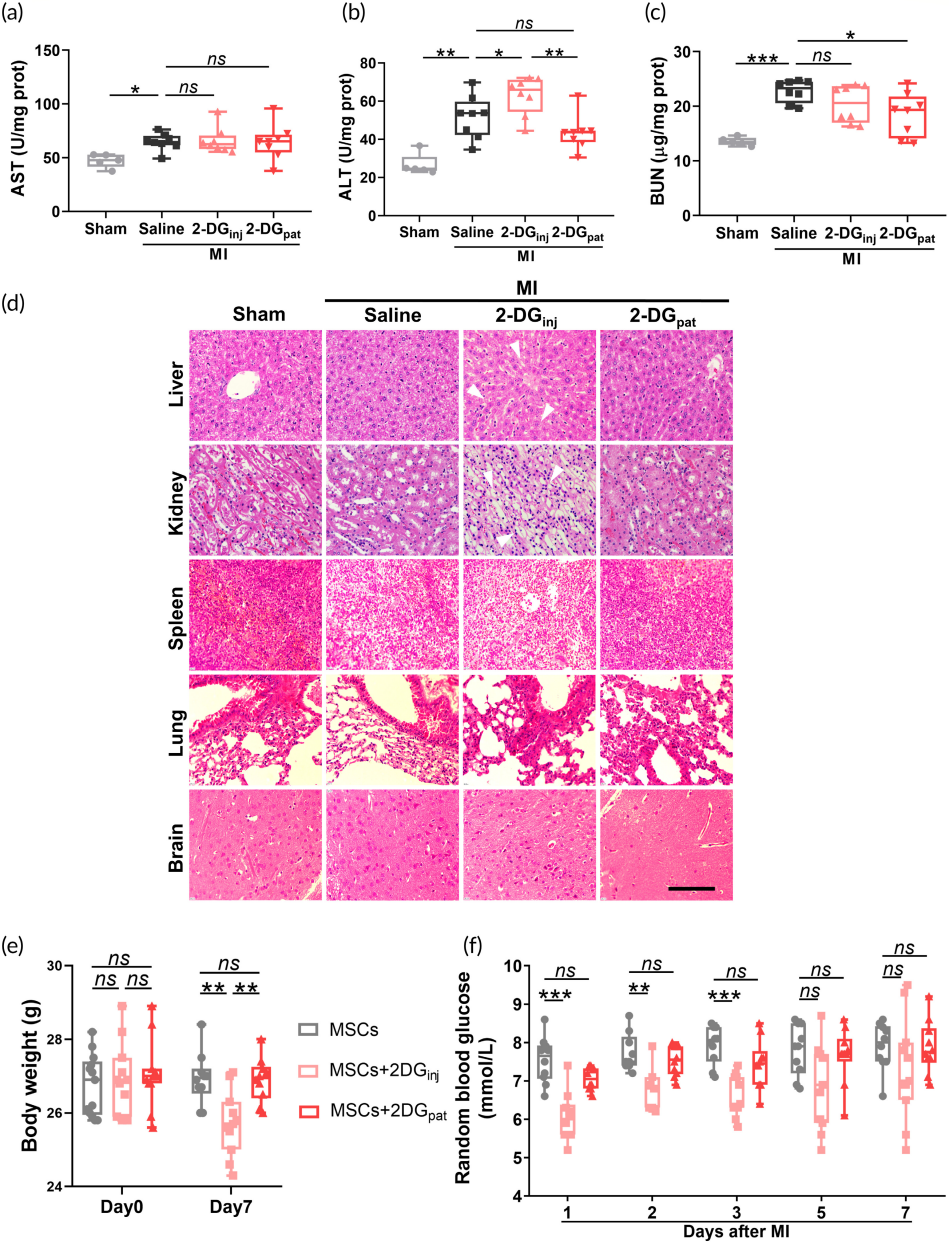
2‐Deoxy‐d‐glucose (2‐DG_pat_) does not induce systemic adverse reactions compared with the 2‐DG intraperitoneal injection. (a–c) Hepatorenal function at 3 days after 2‐DG administration was evaluated by measuring aspartate transaminase (AST) (a), alanine transaminase (ALT) (b), and blood urea nitrogen (BUN) (c) levels (*n* = 5–8). (d) Representative images of hematoxylin and eosin (H&E) staining in the liver, kidney, spleen, lung, and brain on Day 3 after MI. White arrows show vacuolation. Scale bar, 100 μm. (e) Quantitative analysis of body weight on Days 0 and 7 after myocardial infarction (MI) (*n* = 10–11). (f) Random blood glucose levels measured in mice after MI (*n* = 10–11). Error bars represent SD, and significance was determined using one‐way ANOVA followed by Tukey's test (*p < 0.05, **p < 0.01, ***p < 0.001, and *ns* for not significant).

As a metabolic agent, 2‐DG may affect various organs throughout the body. Because the liver and kidney are primary organs responsible for 2‐DG metabolism and excretion, respectively, hepatorenal function was monitored 3 days after MI. Although the aspartate transaminase (AST) levels remained unaltered (Figure 8a), the alanine transaminase (ALT) levels, were significantly increased after LAD ligation and were further aggravated with greater significance in the 2‐DG_inj_ group (Figure 8b), indicating that a systemic injection of 2‐DG induced serious hepatic toxicity. Notably, this toxicity was absent in the 2‐DG_pat_ group (*p* = 0.357) (Figure 8b). Intriguingly, 2‐DG_pat_, but not 2‐DG_inj_, mitigated the MI‐induced increase in blood urea nitrogen (BUN) levels (Figure 8c). We then performed H&E staining of liver, kidney, spleen, lung, and brain sections and observed characteristic vacuolation in liver and kidney following 2‐DG injection, which was absent in the 2‐DG_pat_ group, as evidenced by the merely slight pathological manifestations (Figure 8d and Figure S5). No detectable vacuolation was observed in spleen, lung, and brain tissue. Consistent with the aforementioned systemic toxicity, body weight was also slightly lower in the 2‐DG_inj_ group, whereas this weight loss was inconspicuous in the 2‐DG_pat_ group (*p* = 0.997) (Figure 8e). Surprisingly, as a glycolytic inhibitor, the systemic injection of 2‐DG resulted in transient hypoglycemia, which may return to normal when the injection was withdrawn, whereas 2‐DG_pat_ did not cause blood glucose fluctuations at all (Figure 8f). Besides, different degrees of fatigue and shivering were observed in the 2‐DG_inj_ group (data not shown). In summary, 2‐DG_inj_ unambiguously causes a variety of organ injuries and systemic side effects, which are completely abrogated when 2‐DG_pat_ is adopted.

## 3 DISCUSSION

Current treatments for MI focus on rapid revascularization and reperfusion, including vasodilators, anticoagulants, implantation of stents or bridging vessels, and relief of cardiac burden such as betablockers.^31^ However, these impressive therapies have not yielded satisfactory effect with the mortality and morbidity of complications associated with MI remain high.^1^ In the circumstances, MSCs transplantation has emerged as an innovative and promising tool for their robust potential of paracrine and immunomodulation.^32^ The primary obstacle to MSC therapy is extremely poor retention and low survival, which hampers further clinical application. For decades, a variety of strategies have been devoted to increasing the efficiency of MSC delivery. These strategies include modifying the properties of MSCs, such as increasing the expression of adhesion factors or inflammatory cytokine receptors,^33^, ^34^ preconditioning MSCs with toxic elements, exposure to a hypoxia‐ or nutrient‐free environment,^35^, ^36^ and magnetic targeting techniques.^37^ Meanwhile, emerging studies have focused on the encapsulation of MSCs within biomaterials, including injectable hydrogels,^38^, ^39^ scaffolds,^40^ gelatin coatings,^41^ and microneedles.^42^ Nevertheless, current strategies are limited by the inefficient interactions between implanted cells and excessively inflammatory tissue. Ideal approaches are required not only to deliver more “seeds” (beneficial cells) but also to improve the “soil,” namely, to reduce the hyperinflammatory response in the ischemic region. In this study, 2‐DG application exhibited impressive potential to ameliorate harsh inflammation in the ischemic region (Figure 2), followed by MSC retention extension and cardiac outcome improvement, as expected (Figure 3). The reprogrammed inflammatory response in situ may trigger an escalated effect on the biological behavior of MSCs than previously anticipated.^43^ Therefore, we assumed that 2‐DG facilitates the efficacy of MSC therapy against MI by modulating the immunometabolism of macrophages.

As commonly used biomaterials, the biocompatibility of chitosan and gelatin has been confirmed in previous studies.^44^, ^45^ In the present study, we fabricated a chitosan/gelatin composite patch loaded with 2‐DG. Unlike other biomaterials that encapsulate MSCs to extend their preservation or reinforce their viability, the 2‐DG composite patch directly targets *the infarcted area* to modulate the disturbed inflammatory response, particularly the accumulation of proinflammatory M1 macrophages, which dramatically hinders the retention and viability of engrafted cells. Our study showed superior effects of 2‐DG_pat_ on MSC preservation and myocardial *protection* (Figures 6 and 7). Moreover, this immunometabolic patch slowly released 2‐DG for more than 3 days (Figure 5), the period of peak M1 macrophage accumulation after MI. According to these findings, we infer that 2‐DG_pat_ continuously modulates the immunometabolism of local macrophages and subsequently effectively attenuates hostile hyperinflammation in the infarcted myocardium.

As a typical glycolytic suppressor, 2‐DG has been utilized for decades in many pathological situations, such as tumors^46^ and epilepsy.^47^ Nonetheless, a systemic injection of 2‐DG indeed produces some side effects, including increased morbidity of pheochromocytoma, weight gain loss, unexpected mortality of rodents, and transient hypoglycemia, some of which were also observed in our experiment (Figure 8e,f), even when the dose was reduced to 500 mg/kg/d.^30^ One unanticipated finding was hepatorenal toxicity, as evidenced by increased ALT levels and hepatorenal vacuolation (Figure 8), suggesting that although as it is a small molecule, 2‐DG still exerted a toxic effect on those major metabolic organs. The topical application of 2‐DG_pat_ perfectly evades these side effects and exhibits similar benefits as systemic injection (Figures 6 and 7). These results suggest that in the treatment of local lesions, topical application may be a better choice for the clinical utilization of drugs, especially for those metabolic agents.

One important finding of this study is that macrophage‐dependent attenuation of hyperinflammation is involved in the 2‐DG‐mediated cardioprotective benefits. In the presence of macrophages, MSCs + 2‐DG‐treated MI mice displayed a greater improvement in the retention of MSCs and subsequent cardiac repair. In contrast, after the depletion of macrophages, the protective effect of 2‐DG disappeared completely (Figure 4c–h). When macrophages are absent, 2‐DG loses its target for regulating immune stress, and thus Cl_2_MDP‐lipo‐treated MI mice exhibit aggravated cardiac dysfunction and an increased fibrotic area. Therefore, our purpose is not to obliterate all macrophages or M1 macrophage subsets but to decrease their proinflammatory activation.

In addition, the impact of glycolytic inhibition on MSCs was also explored. Intriguingly, we found that 2‐DG enhanced the gene expression of *Il10*, *Tgfb*, *Vegf*, *Pdgf*, and *Igf‐1* in MSCs under hypoxia (Figure S6), suggesting 2‐DG could not only attenuate the inflammatory response to prolong the MSCs retention but also directly modulate the paracrine of transplanted MSCs, thereby enhancing their potential of pro‐angiogenesis and immunomodulation.

One limitation of the study is the indeterminate energy source of cells or tissues when glycolysis is subdued. Due to limited oxygen and nutrients, glycolysis is important for supplying energy to the myocardium after MI. In a review of the literature, restrained glycolysis induces biosynthesis and alternate fuel consumption,^48^ providing substrates for subsequent biogenesis and energy demand.^49^, ^50^ Further research should be conducted to identify alternate metabolic pathways other than glycolysis, such as amino acid oxidation^48^, ^51^ and ketone body metabolism.^52^, ^53^

## 4 MATERIALS AND METHODS

Detailed materials and methods are in materials and methods section in the Supplementary Data S1.

### 4.1 Cell preparation and treatment

Macrophages were isolated from C57BL/6 mice. Briefly, mice were injected intraperitoneally with 1 ml of starch broth (0.3% yeast powder, 1% peptone, 0.5% sodium chloride, and 5% starch) for 3 consecutive days. Primary mouse peritoneal macrophages were harvested from the peritoneal exudate with precooled RPMI 1640 medium. The cells were incubated at 37°C for 2 h and washed with PBS to remove the nonadherent cells. Macrophages were cultured in RPMI medium containing 10% FBS (Procell) for 24 h and then treated with 2‐DG (1 mM, Sigma‐Aldrich) for 3 h and LPS (100 ng/ml) for another 24 h. The supernatant was collected for pH and lactic acid measurement. Proteins and mRNAs were extracted for subsequent analyses. Bone marrow‐derived MSCs from C57BL/6 mice (Cyagen Biosciences) were cultured in Dulbecco's modified Eagle's medium: F‐12 supplemented with 10% FBS (ExCell Bio) under routine conditions.

### 4.2 Animal study design

For systemic glycolytic inhibition, 62 mice were randomly divided into sham, saline, and 2‐DG group. For synergistic effect of MSC therapy with 2‐DG injection, 35 mice were randomized into saline, MSCs, and MSCs + 2‐DG group. Meanwhile, to compare effect of 2‐DG_pat_ with 2‐DG_inj_, 35 mice were randomly divided into MSCs, MSCs + 2‐DG_inj_, and MSCs + 2‐DG_pat_ group. Moreover, MSCs retention was monitored by CM‐DiI labeling (*n* = 12) and bioluminescence imaging (*n* = 15). For macrophage depletion, 49 mice were randomly divided into four groups: (1) MSCs + PBS‐lipo, (2) MSCs + 2‐DG + PBS‐lipo, (3) MSCs + Cl_2_MDP‐lipo, and (4) MSCs + 2‐DG + Cl_2_MDP‐lipo. Finally, for potential 2‐DG‐associated side effects, 60 mice were randomly divided into four groups: (1) sham, (2) saline, (3) 2‐DG_inj_, and (4) 2‐DG_pat_. Hepatorenal function was detected on Day 3 after MI, and body weight and random blood glucose were recorded.

### 4.3 Preparation and functional assay of the chitosan/gelatin‐based 2‐DG patch

The filter‐sterilized 4% chitosan (w/v, in 1% acetic acid, Senopharm) and 2% gelatin (w/v, in 1% acetic acid, Senopharm) solutions were mixed at a weight ratio of 1:1, and then 1% 2‐DG was added. After complete dissolution by stirring, the impurities and bubbles were removed by centrifugation at 3000–4000 rpm for 15 min. The mixture was poured on a culture plate and placed in a drying oven overnight at 50–66°C, followed by deacidification with 2% NaOH‐80% ethanol. Finally, deionized water was added to remove the excess acetic acid.

To explore the sustained release of 2‐DG_pat_, ddH2O was added to the patch, and the supernatant after soaking were collected continuously for 3 days. The concentration of 2‐DG in the supernatant was detected with detection kit (Solarbio). Meanwhile, RPMI containing 10% FBS was added to the patch, and the supernatant collected 72 h after soaking was denoted as CM to assess the potential function of 2‐DG_pat_. CM was used to treat macrophages for 24 h in the presence or absence of LPS.

### 4.4 MI and 2‐DG administration

All animal procedures were performed according to the *Guide for the Care and Use of Laboratory Animals* (NIH, 8th edition, 2011). MI was established on male C57BL/6 mice (8–10 weeks old) as we previously described.^54^ Briefly, after general anesthesia, LAD was ligated about 2 mm below the lowest part of the left atrial appendage. Successful ligation was verified by pale left ventricular wall below the ligation site. Next, 5 × 10^5^ MSCs in 20 μl of saline were injected intramyocardially at three different sites surrounding the infarct zones. Mice in the sham group underwent only thoracotomy without LAD ligation.

In the 2‐DG injection group (2‐DG_inj_), 500 mg/kg 2‐DG was administered intraperitoneally 6 h before MI and 1 and 2 days after MI. For 2‐DG_pat_ group, a prepared patch was attached to infarct area with fibrin glue (Sigma‐Aldrich) immediately after LAD ligation. The patch size was approximately 3.5 mm in diameter.

### 4.5 Echocardiography

Cardiac function was continuously measured by echocardiography before and after MI induction as we previously described.^55^ Briefly, after inhaled anesthesia, satisfactory two‐dimensional long‐ and short‐axis images of the left ventricle were obtained. The M‐mode view of the parasternal short axis was recorded for analysis. All measurements and analyses were conducted by an experienced researcher who was blinded to the study groups. All results were averaged over three separate cardiac cycles.

### 4.6 Histological analysis

Ischemic hearts were fixed with 4% paraformaldehyde and sectioned. Sections perpendicular to the axis of the LAD were stained with Masson's trichrome (Solarbio) and H&E (Solarbio). Histological images were visualized using a stereoscopic microscope and analyzed with ImageJ software.

### 4.7 Statistical analysis

Statistical significance was analyzed using either SPSS 25.0 or GraphPad Prism 8 software. The normal distribution of data was tested using Shapiro–Wilk test. Variables normally distributed are presented as the means ± standard deviations. Differences between two groups were assessed using Student's *t*‐test. Statistically significant differences among multiple groups were determined using ANOVA with Tukey's test. For the data which was not normally distributed and/or with unequal variance, nonparametric Kruskal–Wallis test was used followed by Dunn's multiple comparison test. For the analysis of cardiac function in multiple groups over time, two‐way repeated measures ANOVA was performed followed by Tukey's test. Differences were considered statistically significant at *p* < 0.05.

## 5 CONCLUSIONS

Distinct from most strategies attempting to optimize MSCs potency or their resistance to hostile environments, in this study, we developed a 2‐DG‐loaded, chitosan/gelatin‐based immunometabolic patch, aiming to tender aggravated glycolysis in ischemic region. On one hand, sustained release of 2‐DG by the patch calmed hyperinflammatory response in ischemic myocardium, thereby facilitating retention of implanted MSCs and enhancing cardiac healing ultimately. On the other hand, topical release of 2‐DG by the patch also avoided potential side effects associated with whole‐body inhibition of glycolysis. In summary, our data support the promise of immunometabolic patch as a novel strategy to optimize cell therapy for treating MI.

## AUTHOR CONTRIBUTIONS

**Weizhang Xiao:** Conceptualization (lead); formal analysis (lead); investigation (lead); methodology (lead); writing – original draft (lead); writing – review and editing (equal). **Ming Chen:** Data curation (equal); formal analysis (equal); investigation (equal); methodology (equal). **Wenjing Zhou:** Data curation (equal); software (equal); validation (equal); visualization (equal). **Liang Ding:** Data curation (equal); software (equal); validation (equal); visualization (equal). **Ziying Yang:** Software (equal); validation (equal); visualization (equal). **Lianbo Shao:** Software (equal); validation (equal); visualization (equal). **Jingjing Li:** Software (equal); validation (equal); visualization (equal). **Weiqian Chen:** Conceptualization (lead); project administration (equal); supervision (lead); writing – review and editing (lead). **Zhenya Shen:** Funding acquisition (lead); project administration (lead); resources (lead).

## CONFLICT OF INTEREST

The authors have no conflicts of interest to declare.

### PEER REVIEW

The peer review history for this article is available at https://publons.com/publon/10.1002/btm2.10471.

## Supporting information


**Data S1:** Supporting Information

## Data Availability

The data that support the findings of this study are available from the corresponding author upon reasonable request.
