# Supplementary material for: An immunometabolic patch facilitates mesenchymal stromal/stem cell therapy for myocardial infarction through a macrophage‐dependent mechanism
Source: Bioeng Transl Med. 2022 Dec 13;8(3):e10471. doi: 10.1002/btm2.10471 (PMC10189442; doi:10.1002/btm2.10471)
Supplement: Supplementary file 1 — Data S1: Supporting Information [file BTM2-8-e10471-s001.docx]

*Title*: An immunometabolic patch facilitates mesenchymal stromal/stem cell therapy for myocardial infarction through a macrophage-dependent mechanism

*Authors*

Weizhang Xiao^#^ (xwz191201@163.com), Ming Chen^#^ (13862557832@163.com), Wenjing Zhou (zhouwenjing0319@163.com), Liang Ding (2453878571@qq.com), Ziying Yang (skyinger@163.com), Lianbo Shao (shaolianbo1987@126.com), Jingjing Li (lijj@suda.edu.cn), Weiqian Chen^*^ (chenweiqian@suda.edu.cn), Zhenya Shen^*^ (uuzyshen@aliyun.com)

**Methods**

**Extracellular acidification rate (ECAR) measurement**

Myeloid ECAR was determined using a glycolysis assay kit (Abcam) to detect glycolytic flux as described in our previous study, with some modifications ^1^. Briefly, macrophages were seeded in a 96-well plate at a density of 3 × 10^5^ cells/well and incubated overnight. Then, the macrophages were stimulated with LPS for 24 h with or without 2-DG pretreatment. Afterward, the culture plates were transferred into a CO_2_-free incubator to purge the media of CO_2_. Culture media were removed from all assay wells, and cells were washed with 100 μL of respiration buffer twice. Another 150 μL of respiration buffer were added to all wells, followed by the addition of 10 μL of reconstituted glycolysis assay reagent to each sample well and 10 μL of respiration buffer to blank control well. The prepared 96-well plate was placed in a fluorescence plate reader (BIO-TEK) preset to 37 °C. The glycolysis assay signal was recorded for 180 min with a delay time of 20 μs and integration time of 100 μs at 1.5 min intervals using excitation and emission (Ex/Em) wavelengths of 380/615 nm.

**Characterization of murine bone marrow-derived MSCs**

The immunophenotype of MSCs was characterized by flow cytometry (Millipore Guava Easycyte) with antibodies against mouse Sca-1-APC, CD44-APC, CD29-FITC, CD45-PE, CD11b-APC, CD117-APC, and their relative isotype controls (MultiSciences), as we previously described ^2^. The osteogenic, chondrogenic and adipogenic differentiation potential of MSCs were evaluated using corresponding differentiation mediums (Cyagen Biosciences).

**Labeling of murine bone marrow-derived MSCs**

Bone marrow-derived MSCs from C57BL/6 mice (Cyagen Biosciences) were labeled with 2 μg/mL chloromethylbenzamido-DiI (CM-DiI, Invitrogen) according to the manufacturer’s instructions. A lentivirus containing firefly luciferase (GeneChem) was transduced into MSCs (Fluc-MSCs). Luciferase activity was determined with a Firefly Luciferase Reporter Gene Assay Kit (Beyotime).

**Selective macrophage depletion**

Selective macrophage depletion was induced via intraperitoneal injection of 60 μL of clodronate-liposomes (Cl_2_MDP, Liposoma BV) 1 day prior to and 1 day after MI. PBS-lipo was injected as control.

**Scanning electron microscopy**

The freeze-dried samples were adhered onto conductive tape and sputtered with gold to observe the morphology of the composite patch. The microstructure of the patch was examined using a scanning electron microscope (SEM, S-4800, Hitachi).

**Immunofluorescence staining**

The immunofluorescence procedure was conducted as we previously described ^3^. Briefly, cells or heart sections were incubated with anti-iNOS (Proteintech), anti-F4/80 (Abcam), or anti-cTnT (Abcam) at 4 °C overnight, followed by fluorescently labeled secondary antibodies. Nuclei were counterstained with DAPI (Meilun Biotechnology Co., Ltd). Images were captured with a confocal microscope and analyzed with ImageJ software.

**Preparation of cardiac single-cell suspensions and flow cytometry analysis**

Cardiac single-cell suspension was prepared as described previously, with slight modifications ^4^. Briefly, mice were anesthetized and intracardially perfused with PBS to eliminate blood cells. The hearts were excised, minced with fine scissors, and digested in a Hanks’ balanced salt solution-based enzyme solution containing a cocktail of 1 mg/mL type II collagenase (Sigma–Aldrich), 0.5 mg/mL dispase (Sigma–Aldrich), 0.5 mM CaCl_2_, and 1 U/mL DNase I (Sigma–Aldrich) for 1 h at 37 °C with oscillation every 15 min. After digestion, samples were filtered with a 70-μm cell strainer. Cells used in subsequent analyses were resuspended and counted after erythrocyte lysis.

For the analysis of cell surface markers, a single-cell suspension was incubated with fluorescently labeled antibodies against cell surface antigens (anti-F4/80-FITC, Invitrogen) at RT for 30 min. For intracellular marker detection, a Foxp3 fixation/permeation working solution (Invitrogen) was first added at RT for 1 hour, and cells were incubated with anti-iNOS-PE (Invitrogen) or anti-CD206-APC (Invitrogen) for 30 min. Cells were washed and resuspended for flow cytometry (Millipore Guava Easycyte), as we previously described ^5^.. All flowcytometry gates and isotype control were presented in the supplementary data.

**Bioluminescence imaging in vitro and in vivo**

Bioluminescence imaging (BLI) was performed using an IVIS Lumina XR III imaging system (Perkin Elmer). Different amounts of cells were incubated in medium containing 150 μg/mL D-luciferin (Yeasen) for 5 min at 37 °C, followed by image capture. For *in vivo* BLI, mice were received an intraperitoneal injection of D-luciferin (150 mg/kg body weight). BLI was recorded continuously for 10 min at 1-min intervals until the signal culminated. BLI signals in the region of interest (ROI) were investigated.

**Superoxide,** **alanine transaminase, aspartate transaminase, and blood urea nitrogen measurement**

Local superoxide level in ischemic myocardium was determined by dihydroethidium (DHE, Beyotime) staining as we described previously ^6^. Heart sections were incubated with 10 μM DHE for 30 min in the dark and then gently washed away with PBS. The red DHE signal was collected using an inverted fluorescence microscope. Alanine transaminase (ALT), aspartate transaminase (AST), and blood urea nitrogen (BUN) levels were determined with corresponding detection kits (Solarbio).

**Quantitative real-time polymerase chain reaction (qRT–PCR)**

Total RNA was extracted with TRIzol (Invitrogen). 500 ng of RNA were reverse transcribed into cDNAs using the Takara PrimeScript RT Reagent Kit. Quantitative real-time PCR was performed with SYBR Premix Ex Taq reaction mix (Takara) on StepOne Plus System (Applied Biosystems) as we previously described ^7^. Gene expression relative to *18S* RNA was analyzed using the 2^-ΔΔCT^ method.

**Western blot**

Routine Western blotting was performed as we previously described ^8^. Briefly, total protein was collected with RIPA buffer and quantitated with a BCA assay kit (Novoprotein). Equal amounts of protein were subjected to SDS-polyacrylamide gel electrophoresis and transferred to polyvinylidene difluoride membranes. The membranes were then incubated with anti-iNOS (Proteintech), anti-IL-1β (Abcam) or anti-β-tubulin (Santa Cruz) overnight at 4 °C. After incubation with peroxidase-conjugated secondary antibodies (Cell Signaling Technology), protein signals were visualized using enhanced chemiluminescence (Vazyme).


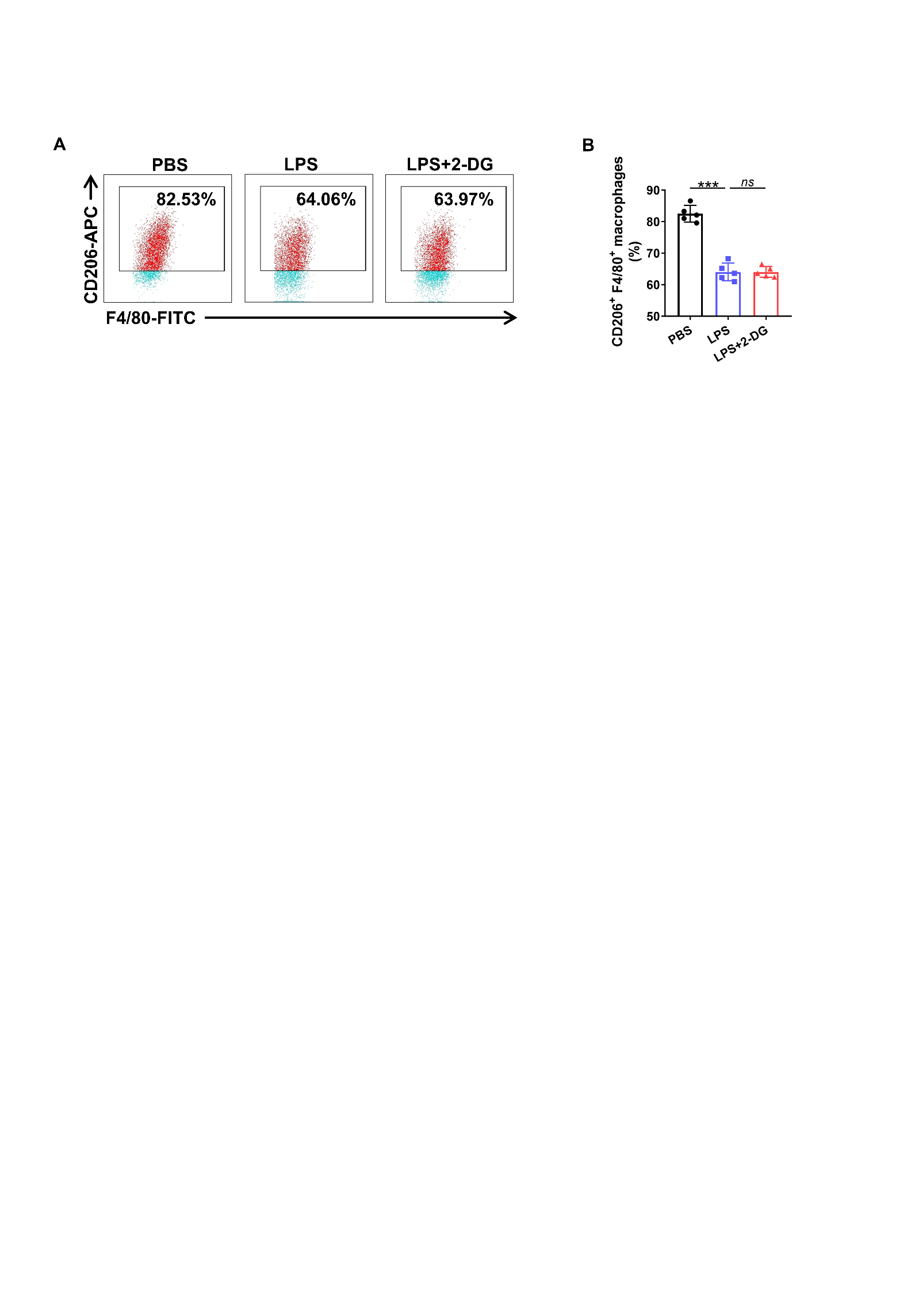


**Fig. S1.** Glycolytic inhibition does not affect the anti-inflammatory polarization of macrophages. (A and B) Representative flow cytometry plots (A) and quantification (B) of the CD206^+^ F4/80^+^ macrophage population (n = 5). Error bars represent the SD, and significance was determined using one-way ANOVA followed by Tukey’s test (****p* <0.001, *ns* for not significant).


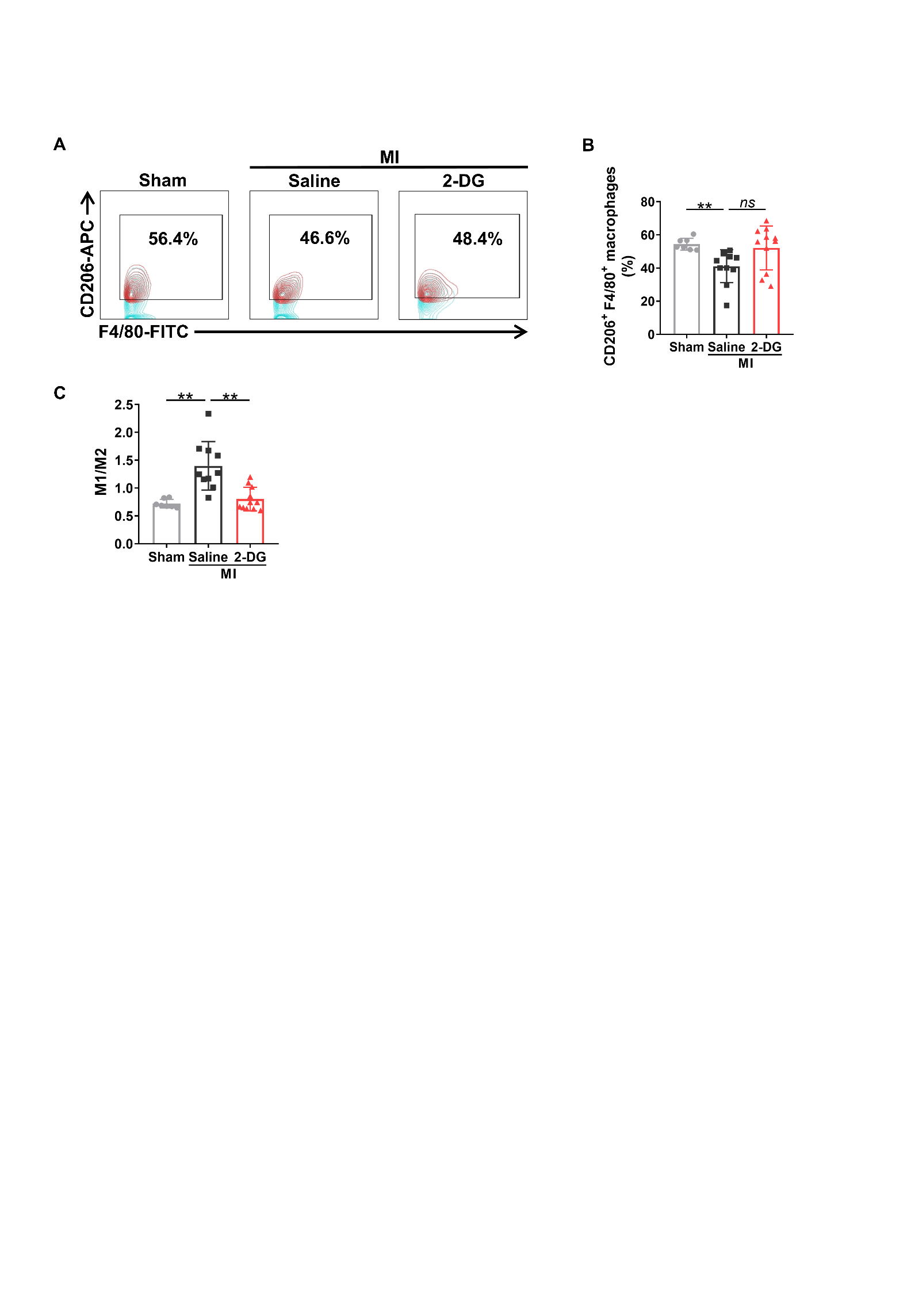


**Fig. S2.** 2-DG failed to promote the anti-inflammatory polarization of macrophages in infarcted myocardium. (A and B) Representative flow cytometry plots (A) and quantification (B) of the CD206^+^ F4/80^+^ macrophage population (n = 7-11). (C) The M1/M2 ratio in ischemic hearts (n = 7-11). Error bars represent the SD, and significance was determined using one-way ANOVA followed by Tukey’s test (***p* < 0.01, *ns* for not significant).


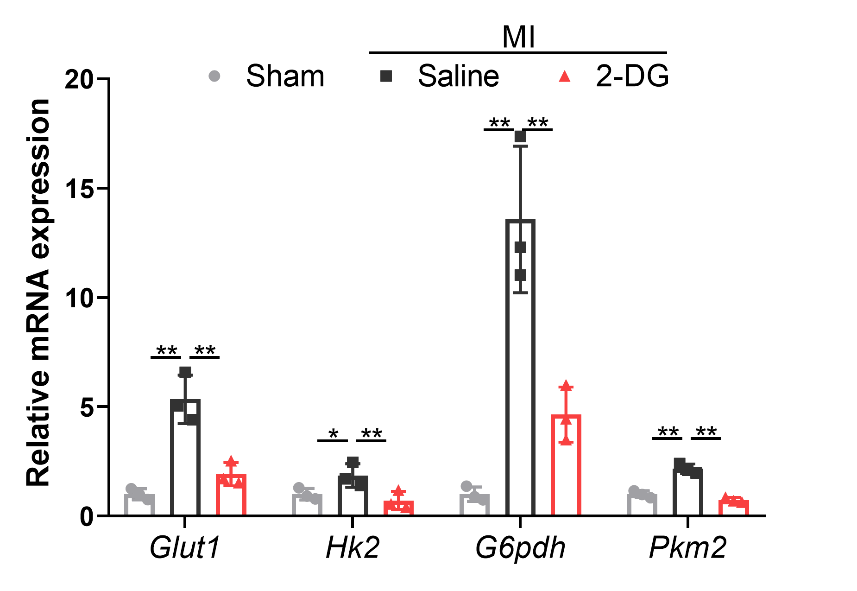


**Fig. S3.** 2-DG inhibits glycolysis in ischemic region post-MI. Gene expression of glycolytic rate-limiting enzymes *Glut1*, *Hk2*, *G6pdh,* and *Pkm2* in infarcted area (n = 3) 3 days after MI by Real-time PCR. Data are presented as mean ± SD. Statistical difference was determined by one-way ANOVA followed by Turkey test (**p* < 0.05, and ***p* < 0.01).


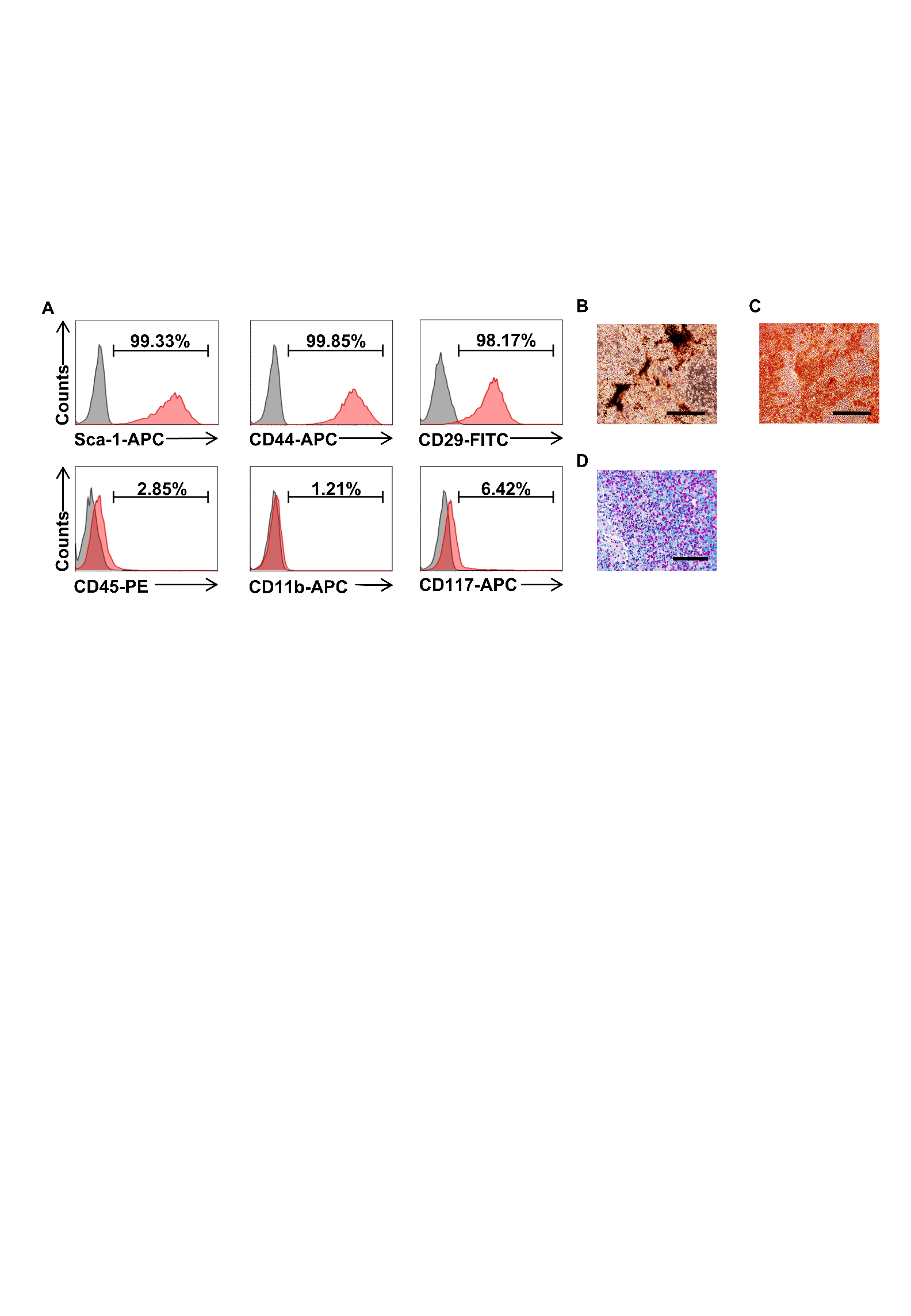


**Fig. S4.** Identification of MSCs. (A) Flow cytometry analysis of cell surface antigens for MSCs. Most MSCs express Sca-1, CD44, CD29, and they do not express CD45, CD11b, and CD117. (B-D) Representative graph of adipogenesis, osteogenesis, and chondrogenesis of MSCs. Scale bar, B, 400 μm; C, 400 μm; D, 50 μm.


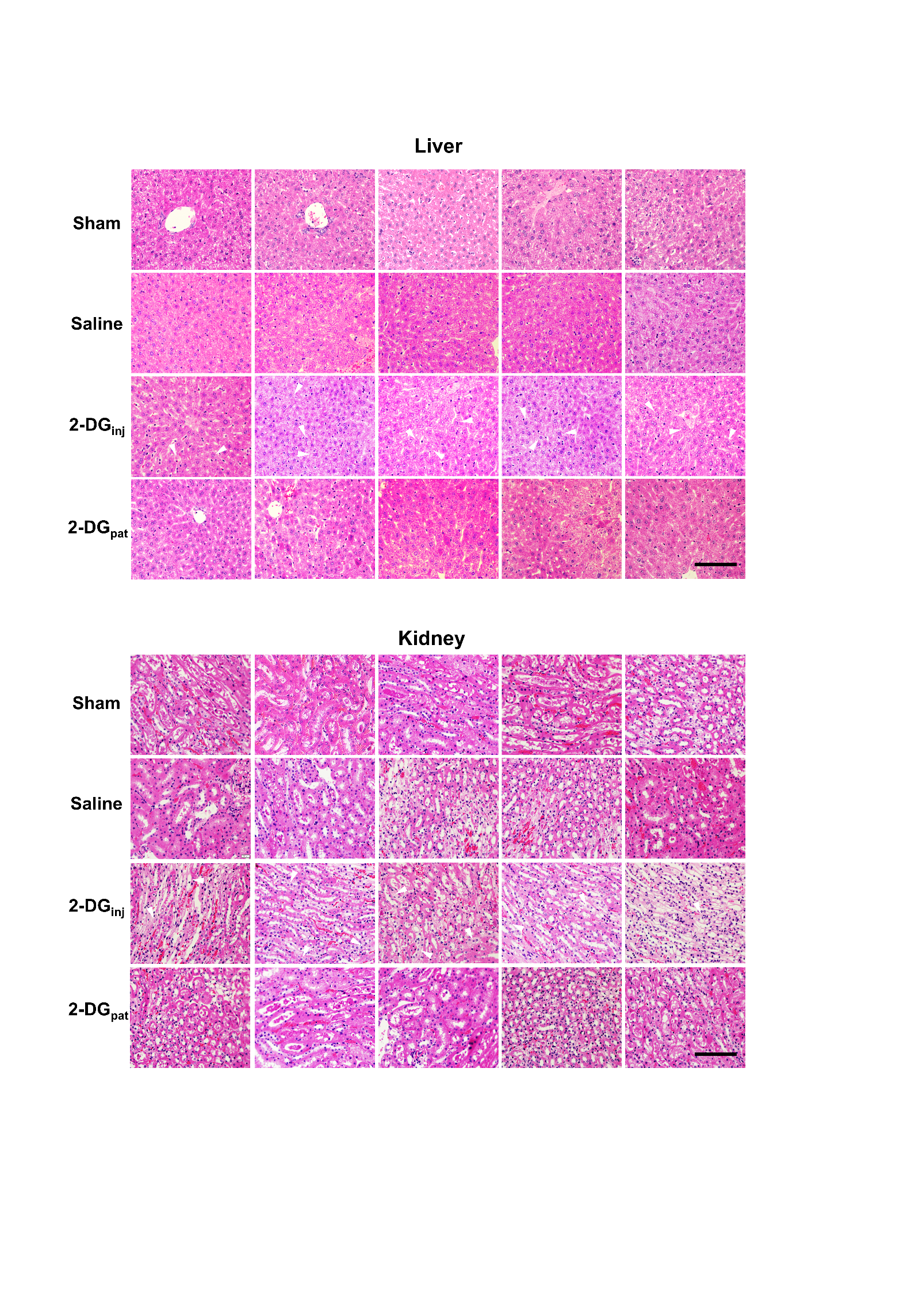


**Fig. S5.** 2-DG_pat_ does not induce systemic adverse reactions compared with the 2-DG intraperitoneal injection. Representative images of H&E staining in liver and kidney on Day 3 after MI. White arrows show vacuolation. Scale bar, 100 μm.


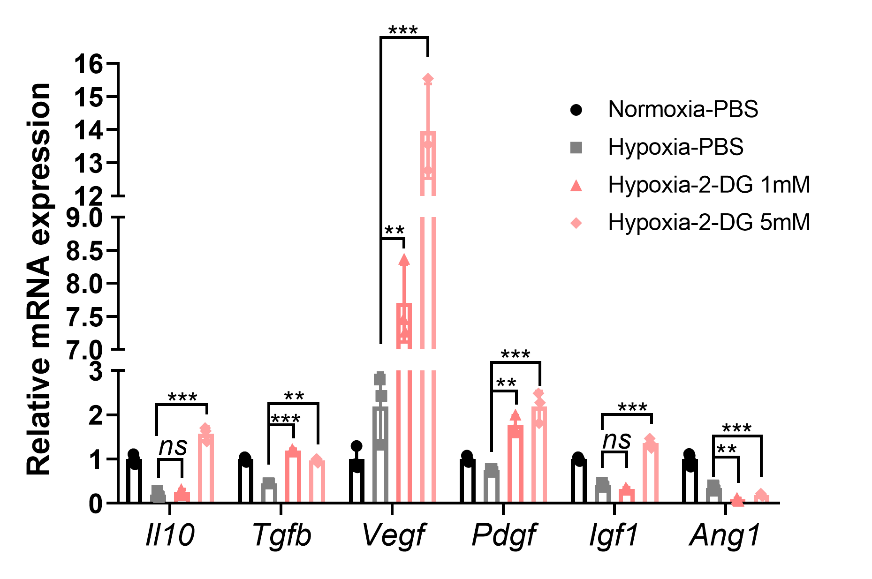


**Fig. S6.** 2-DG modulates the paracrine of MSCs. Gene expression of *Il10*, *Tgfb*, *Vegf*, *Pdgf*, *Igf1* and *Ang1* in MSCs under hypoxia (n = 3) by Real-time PCR. Data are presented as mean ± SD. Statistical difference was determined by one-way ANOVA followed by Turkey test (***p* < 0.01, ****p* <0.001, and *ns* for not significant).

**Flowcytometry gate**


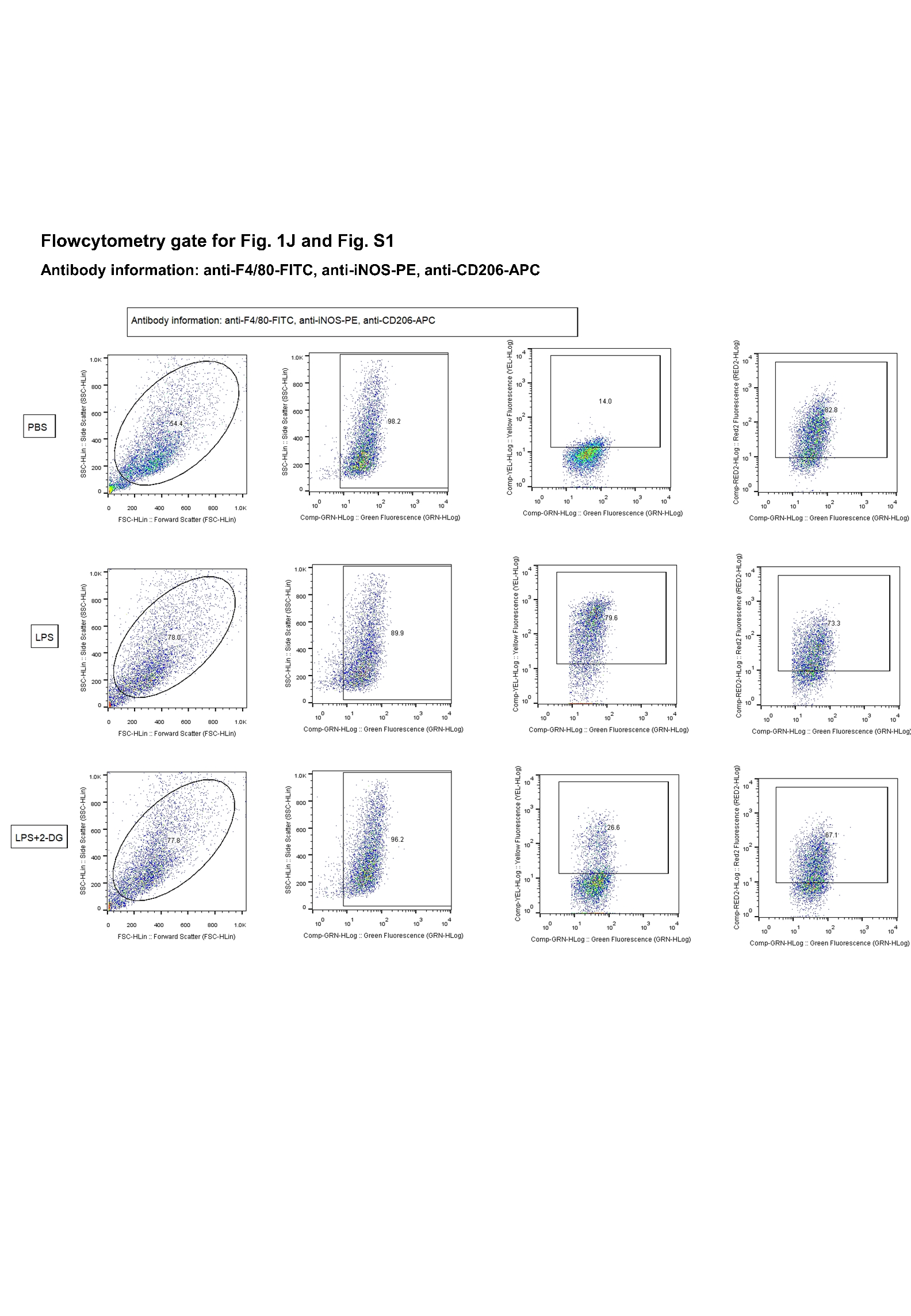


**Flowcytometry gate**


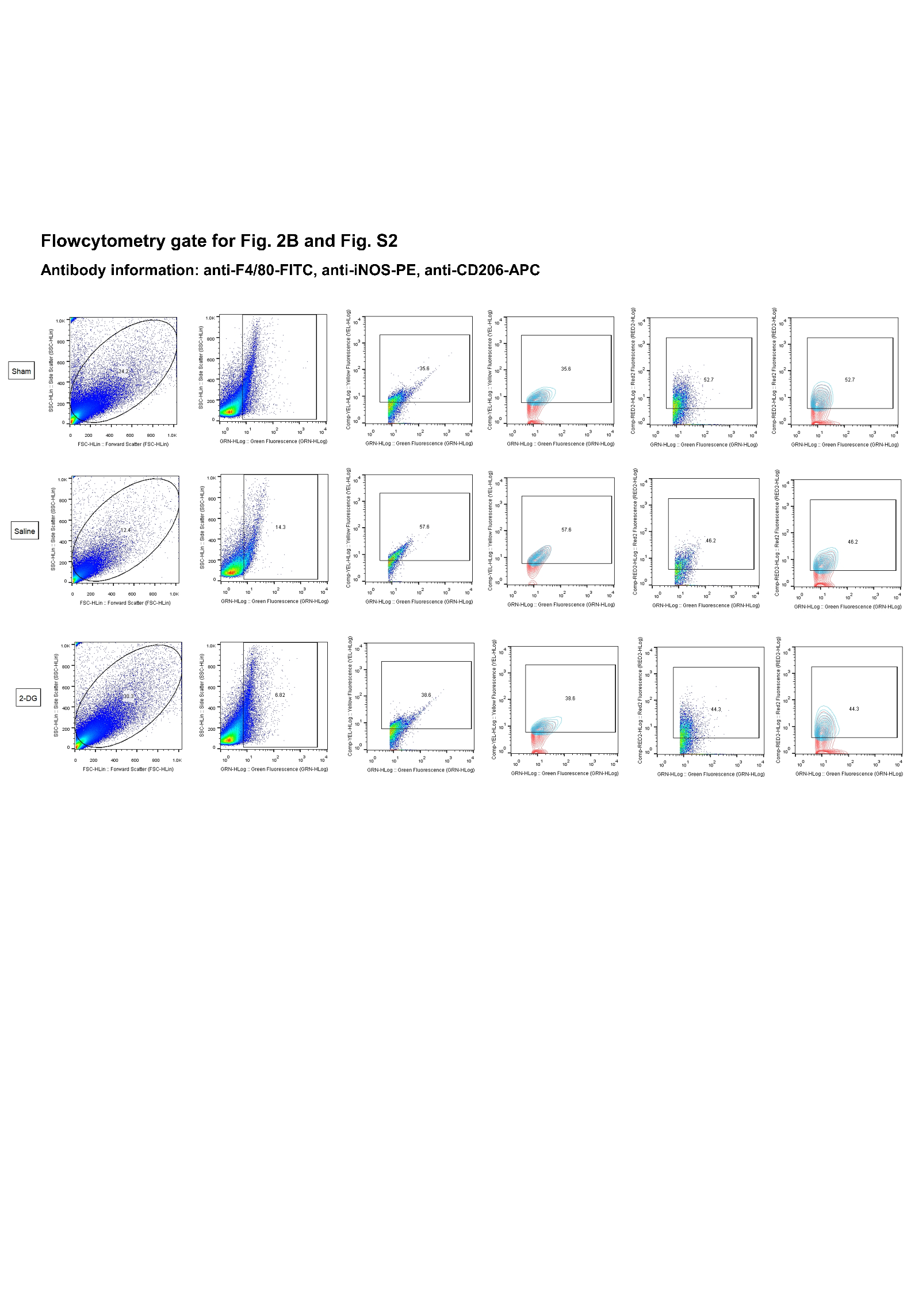


**Flowcytometry gate**


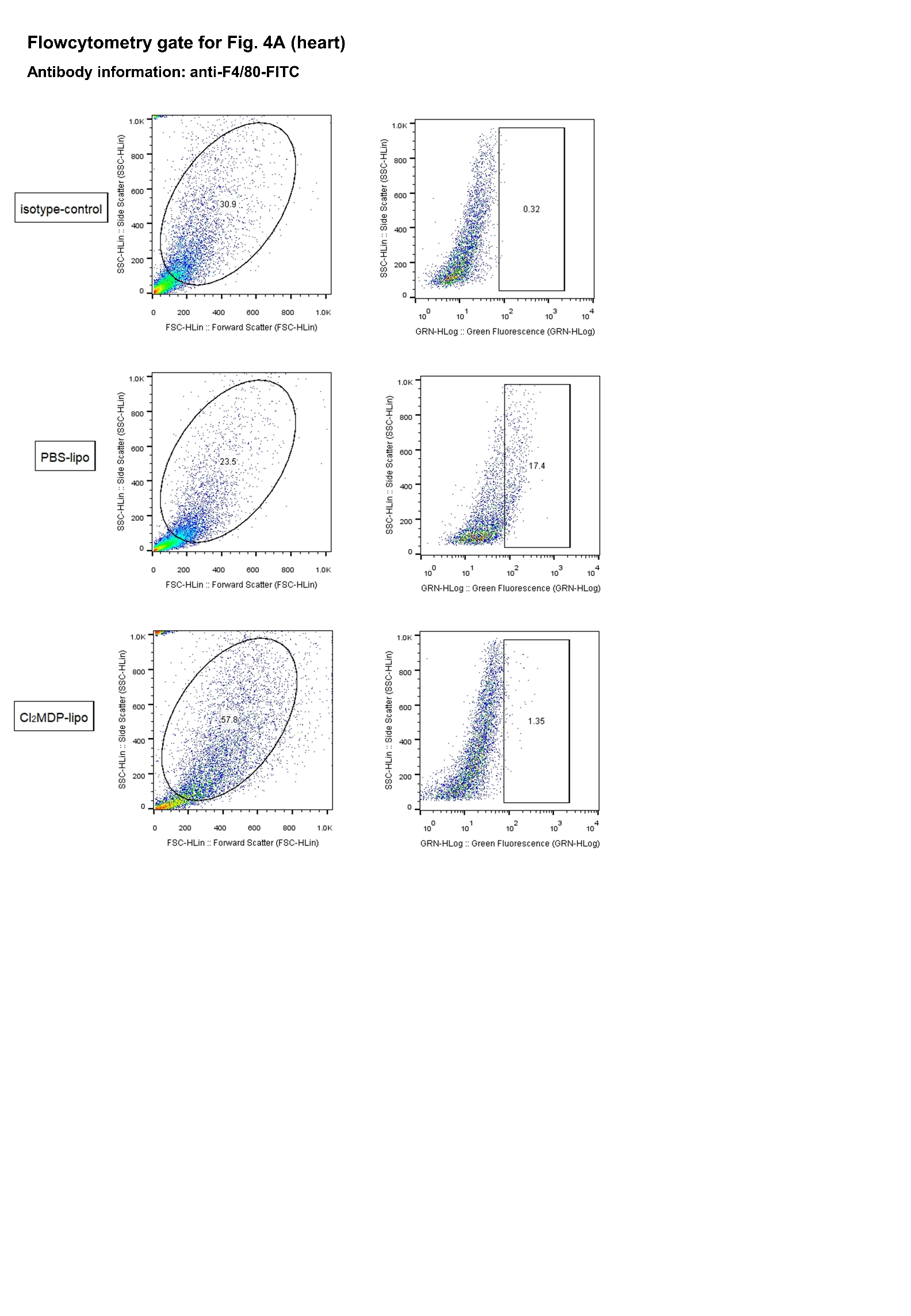


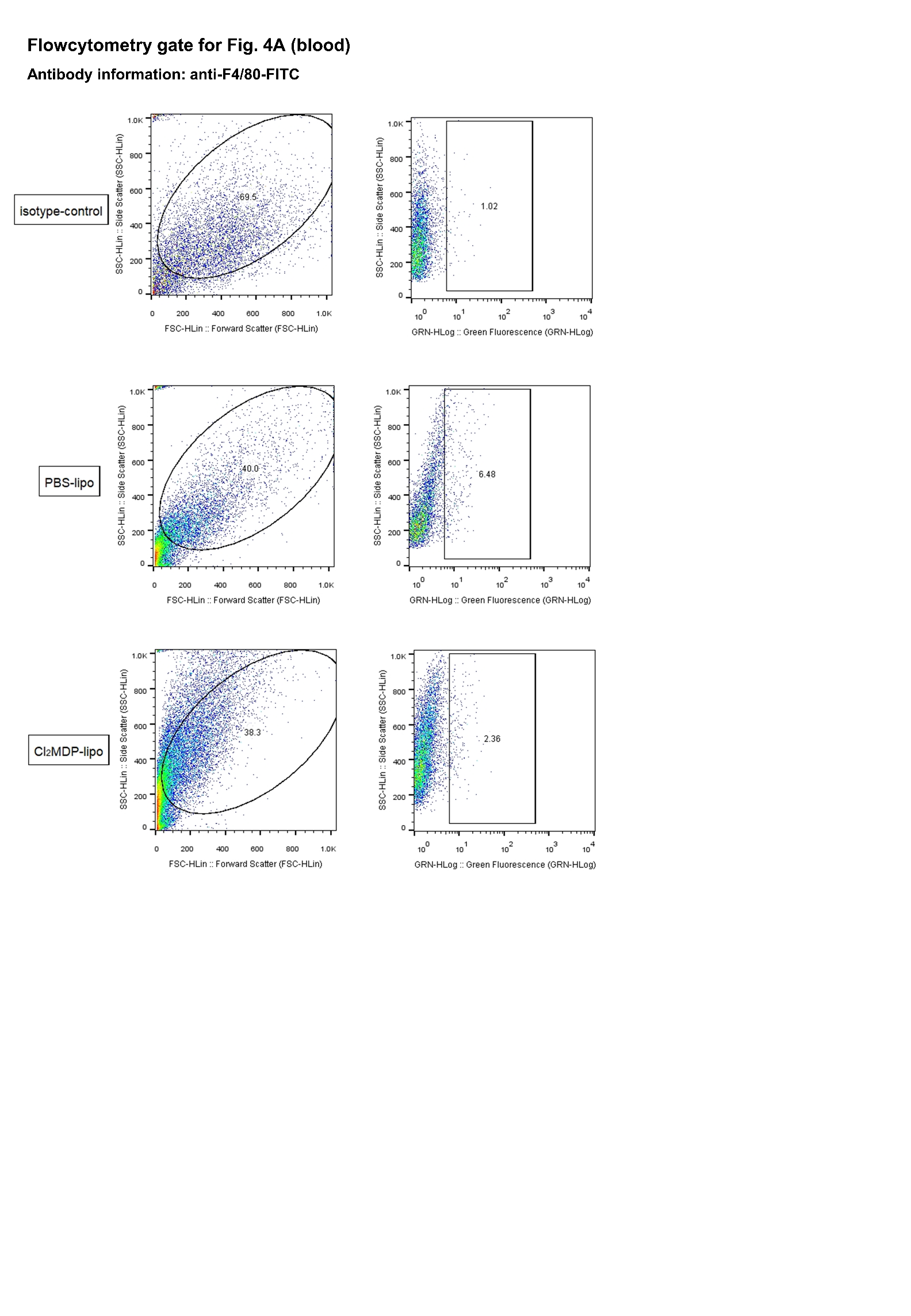


、
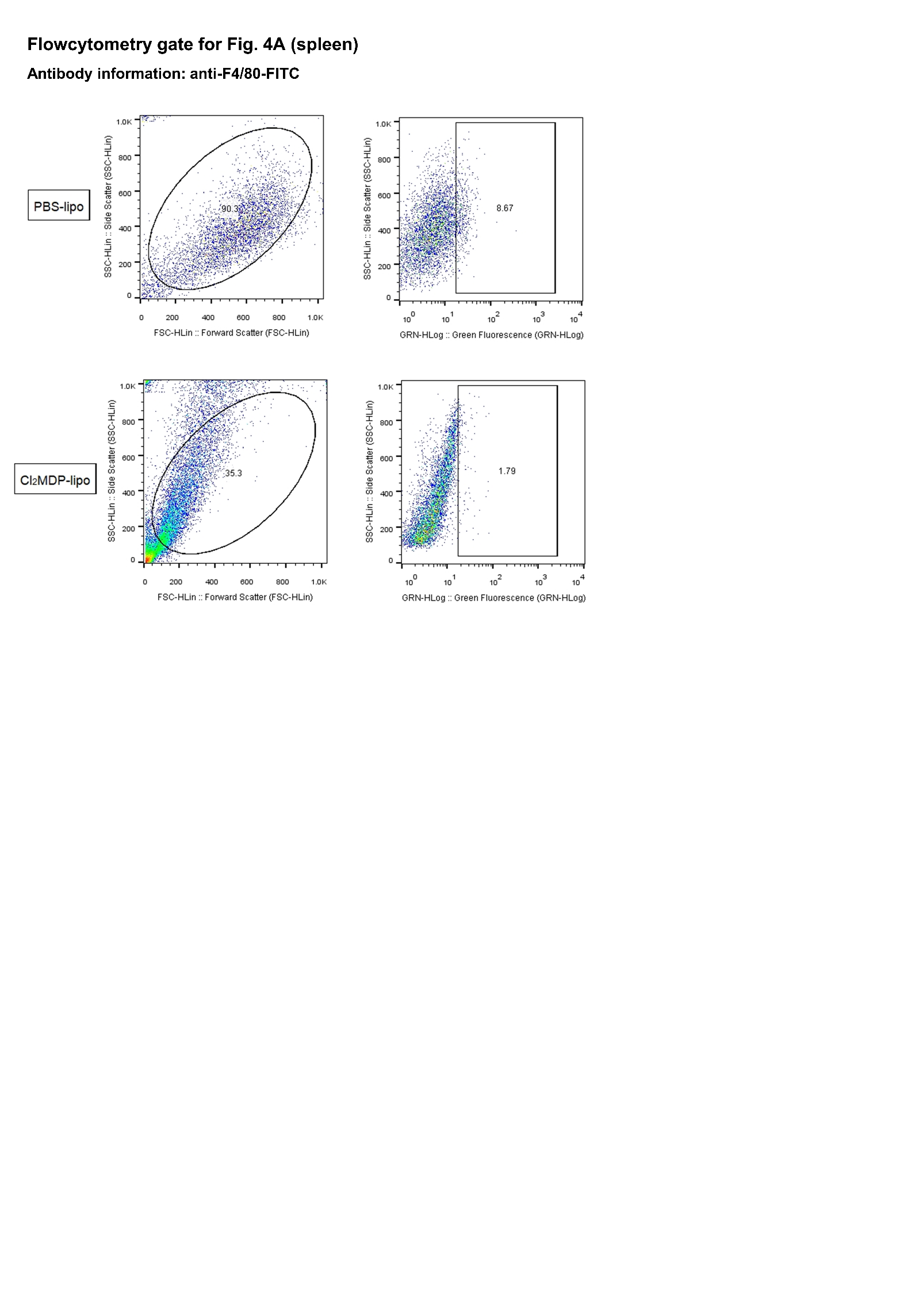


**Flowcytometry gate**

**
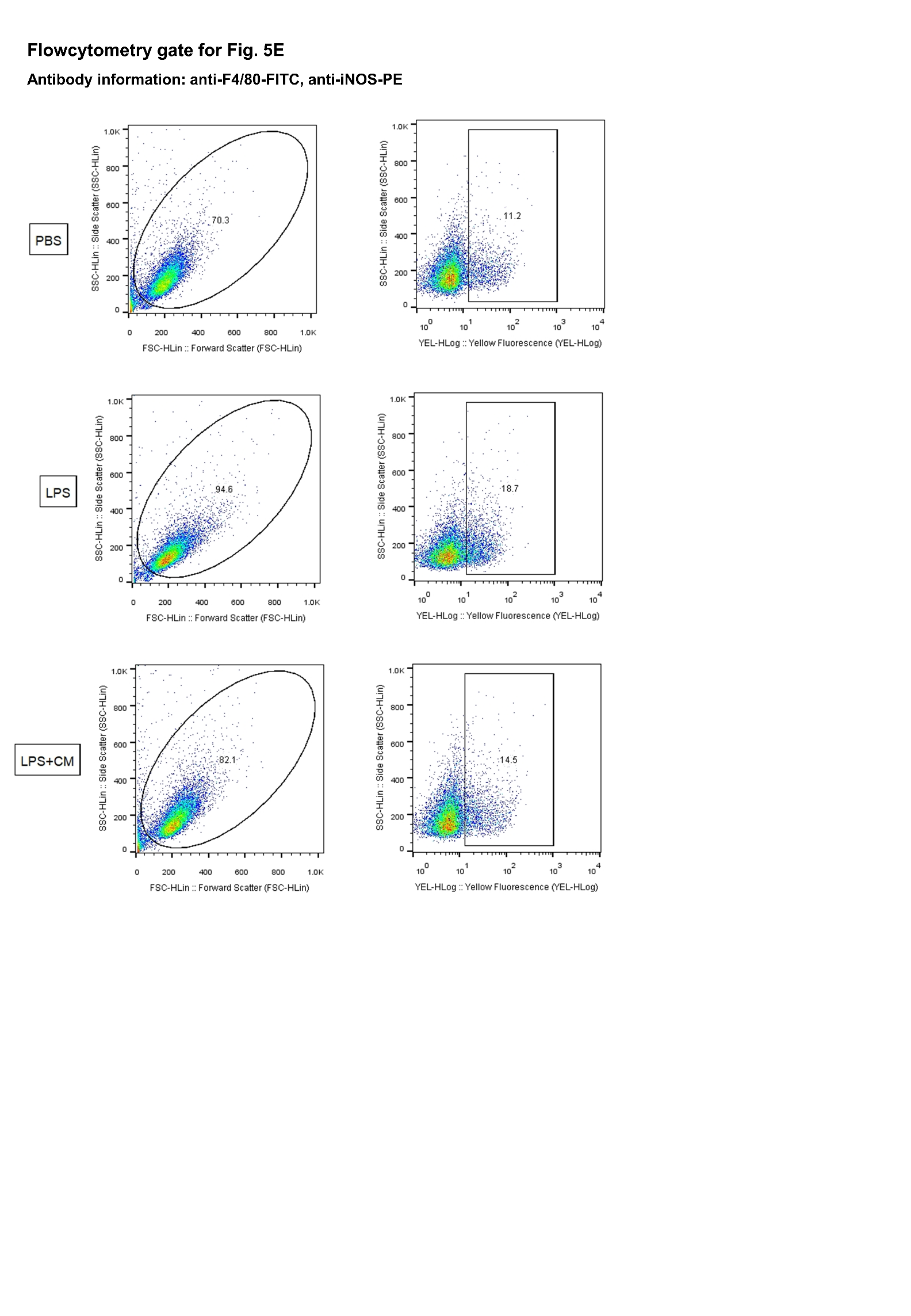
**

**References**

1. Zhao P, Zhou W, Zhang Y, Li J, Zhao Y, Pan L, Shen Z, Chen W, Hui J. Aminooxyacetic acid attenuates post-infarct cardiac dysfunction by balancing macrophage polarization through modulating macrophage metabolism in mice. *J Cell Mol Med*. 2020;24:2593-2609.

2. Wang X, Chen Y, Zhao Z, Meng Q, Yu Y, Sun J, Yang Z, Chen Y, Li J, Ma T, Liu H, Li Z, Yang J, Shen Z. Engineered Exosomes With Ischemic Myocardium-Targeting Peptide for Targeted Therapy in Myocardial Infarction. *J Am Heart Assoc*. 2018;7:e008737.

3. Shen H, Cui G, Li Y, Ye W, Sun Y, Zhang Z, Li J, Xu G, Zeng X, Zhang Y, Zhang W, Huang Z, Chen W, Shen Z. Follistatin-like 1 protects mesenchymal stem cells from hypoxic damage and enhances their therapeutic efficacy in a mouse myocardial infarction model. *Stem Cell Res Ther*. 2019;10:17.

4. Fan Q, Tao R, Zhang H, Xie H, Lu L, Wang T, Su M, Hu J, Zhang Q, Chen Q, Iwakura Y, Shen W, Zhang R, Yan X. Dectin-1 Contributes to Myocardial Ischemia/Reperfusion Injury by Regulating Macrophage Polarization and Neutrophil Infiltration. *Circulation*. 2019;139:663-678.

5. Chen W, Gu P, Jiang X, Ruan HB, Li C, Gao X. Protein phosphatase 2A catalytic subunit α (PP2Acα) maintains survival of committed erythroid cells in fetal liver erythropoiesis through the STAT5 pathway. *Am J Pathol*. 2011;178:2333-2343.

6. Xiao Y, Zhang Y, Chen Y, Li J, Zhang Z, Sun Y, Shen H, Zhao Z, Huang Z, Zhang W, Chen W, Shen Z. Inhibition of MicroRNA-9-5p Protects Against Cardiac Remodeling Following Myocardial Infarction in Mice. *Hum Gene Ther*. 2019;30:286-301.

7. Wu J, Wang J, Zeng X, Chen Y, Xia J, Wang S, Huang Z, Chen W, Shen Z. Protein phosphatase 2A regulatory subunit B56β modulates erythroid differentiation. *Biochem Biophys Res Commun*. 2016;478:1179-1184.

8. Wu J, Dong Y, Teng X, Cheng M, Shen Z, Chen W. Follistatin-like 1 attenuates differentiation and survival of erythroid cells through Smad2/3 signaling. *Biochem Biophys Res Commun*. 2015;466:711-716.
